# Novel 1,2,3-Triazole-sulphadiazine-ZnO Hybrids as Potent Antimicrobial Agents against Carbapenem Resistant Bacteria

**DOI:** 10.3390/antibiotics11070916

**Published:** 2022-07-07

**Authors:** Faizah S. Aljohani, Nadjet Rezki, Mohamed R. Aouad, Mohamed Hagar, Basant A. Bakr, Marwa M. Shaaban, Bassma H. Elwakil

**Affiliations:** 1 Department of Chemistry, College of Science, Taibah University, Al-Madinah Al-Munawarah 30002, Saudi Arabia; nadjetrezki@yahoo.fr (N.R.); aouadmohamedreda@yahoo.fr (M.R.A.); 2 Department of Chemistry, Faculty of Science, Alexandria University, Alexandria 21321, Egypt; mohamedhaggar@gmail.com; 3 Department of Zoology, Faculty of Science, Alexandria University, Alexandria 21321, Egypt; bassant.kamal@pua.edu.eg; 4 Department of Pharmaceutical Chemistry, Faculty of Pharmacy, Alexandria University, Alexandria 21521, Egypt; marwa.mamdouh@alexu.edu.eg; 5 Department of Medical Laboratory Technology, Faculty of Applied Health Sciences Technology, Pharos University in Alexandria, Alexandria 21311, Egypt

**Keywords:** sulphadiazine-ZnO hybrids, IMP, NDM, pneumonia, docking, enzyme assay, in vivo

## Abstract

Bacterial pneumonia is considered one of the most virulent diseases with high morbidity and mortality rates, especially in hospitalized patients. Moreover, bacterial resistance increased over the last decades which limited the therapy options to carbapenem antibiotics. Hence, the metallo-β-lactamase-producing bacteria were deliberated as the most deadly and ferocious infectious agents. Sulphadiazine-ZnO hybrids biological activity was explored in vitro and in vivo against metallo-β-lactamases (MBLs) producing *Klebsiella pneumoniae*. Docking studies against NDM-1 and IMP-1 MBLs revealed the superior activity of the 3a compound in inhibiting both MBLs enzymes in a valid reliable docking approach. The MBLs inhibition enzyme assay revealed the remarkable sulphadiazine-ZnO hybrids inhibitory effect against NDM-1 and IMP-1 MBLs. The tested compounds inhibited the enzymes both competitively and noncompetitively. Compound 3b-ZnO showed the highest antibacterial activity against the tested metallo-β-lactamase producers with an inhibition zone (IZ) diameter reaching 43 mm and a minimum inhibitory concentration (MIC) reaching 2 µg/mL. Sulphadiazine-ZnO hybrids were tested for their in vitro cytotoxicity in a normal lung cell line (BEAS-2Bs cell line). Higher cell viability was observed with 3b-ZnO. Biodistribution of the sulphadiazine-ZnO hybrids in the lungs of uninfected rats revealed that both [^124I^]3a-ZnO and [^124I^]3b-ZnO hybrids remained detectable within the rats’ lungs after 24 h of endotracheal aerosolization. Moreover, the residence duration in the lungs of [^124I^]3b-ZnO (t_1/2_ 4.91 h) was 85.3%. The histopathological investigations confirmed that compound 3b-ZnO has significant activity in controlling bacterial pneumonia infection in rats.

## 1. Introduction

*Klebsiella pneumoniae* (Kp) is among the most prevalent Gram-negative bacterial infections in the intensive care units (ICUs) especially the ventilated patients [1]. Kp is a member of the “ESKAPE” microorganisms which includes *Enterococcus faecium*, *Staphylococcus aureus*, *Klebsiella pneumoniae*, *Acinetobacter baumanii*, *Pseudomonas aeruginosa*, and *Enterobacter* spp. [2]. Resistance to antibiotics is an emerging crisis that affects human health [3] that has the possibility of ending the modern medicine era [4]. β-lactam antibiotics are the most extensively used class of antibiotics against human and veterinary diseases [5]. β-lactam antibiotics share a common four-membered ring of β-lactam (the antibiotics’ pharmacophore) [6]. The mounting bacterial resistance towards β-lactam antibiotics is mainly due to the β-lactamases’ production which researchers tried to conquer by designing, synthesizing, and repurposing several β-lactamase inhibitors [7]. β-lactamases were classified according to the amino acid sequences into four molecular classes (A, B, C, and D) [6]. A, C, and D classes were also named serine-β-lactamases (SBLs) which were penicillins, cephalosporins, and monobactams inhibitory enzymes [8]. On the other hand, class B enzymes were known as metallo-β-lactamases (MBLs) which were more virulent enzymes because of their activity against most penicillins, cephalosporins, and carbapenems [9]. MBLs were further classified into three subclasses (B1, B2, and B3), and the enzymes of the B1 subclass (including IMP-1, VIM-2, and NDM-1) have emerged as the most clinically significant subclass [10]. New Delhi metallo-β-lactamase (NDM-1) raises major concerns because of its ability to hydrolyze and inactivate almost all the β-lactam antibiotics [10,11]. Kp can develop extended-spectrum β-lactamase (ESBL), metallo-β-lactamase, or even carbapenemase activity that limits the treatment options [1].

The currently available β-lactamase inhibitors are only temporary solutions to restore β-lactam antibiotics’ activity because they are only active against serine β-lactamases, while metallo-β-lactamases continued to evolve the resistance to all the β-lactam antibiotics including carbapenems [7].

### Rational Study

Sulfonamides, sometimes known as “sulfa medications” are a class of antibiotics used to treat bacterial infections. They may be prescribed to treat bronchitis, eye infections, bacterial meningitis, pneumonia, ear infections, severe burns, traveler’s diarrhea, and other disorders. 

**Figure 1:**
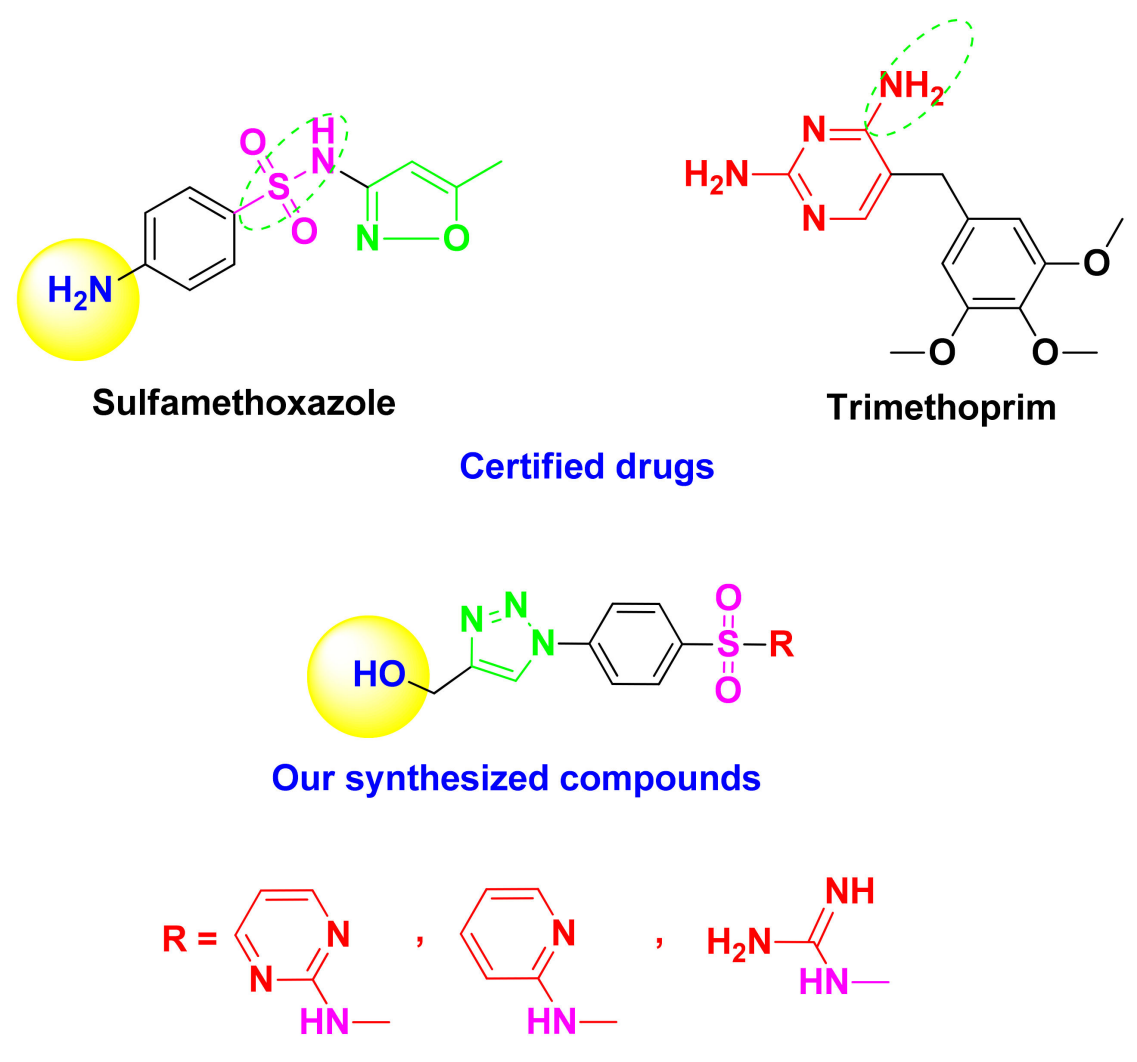
Certified drugs against bacterial pneumonia.

An aminopyrimidine is an antibiotic with pyrimidine 2,4-diamine and 1,2,3-trimethoxybenzene moieties connected by a methylene bridge. The ChEBI team has manually annotated this object. Trimethoprim (TMP) is an antibiotic that is primarily used to treat bladder infections. Moreover, trimethoprim is an antibiotic that is commonly used to treat systemic bacterial infections. It acts by inhibiting bacterial growth. This antibiotic exclusively addresses bacterial illnesses. It is ineffective against viral infections (such as the common cold and flu). When an antibiotic is used when it is not required, it may become ineffective against future illnesses [12] (Figure 1). 

Sulfonamide derivative antibiotics include trimethoprim and sulfamethoxazole. By competing with para-aminobenzoic acid, this drug reduces bacterial production of dihydrofolic acid, consequently decreasing folic acid synthesis and inhibiting bacterial growth. Except for *P. aeruginosa*, TMP-SMZ has antibacterial efficacy against typical urinary tract infections. We targeted our design on mimicking the approved drugs, particularly the most powerful polar groups, which are known to be essential groups in the interaction with the receptor protein via hydrogen bonding, resulting in greater biological activity [13,14].

Nanomaterials have wide applications due to their marvelous functional and structural properties from the parent bulk material [15]. Anticipating the emergence of resistant microbes and making use of the benefits of nanoparticles, researchers have begun to develop new antimicrobial agents using nanomaterials [16]. Inorganic nanoparticles are being promoted as an alternative to traditional antibacterial treatments. From their less widespread use to their high stability, inorganic nanoparticles are being proposed as an alternative antibacterial agent for MDROs for a variety of reasons [17]. MBLs require one or two zinc ions for their activity, these ions are essential for substrate binding as well as enzyme catalysis. It was observed that MBLs respond differently to the presence of various concentrations of zinc ions. In addition, a certain range of zinc concentrations was required to produce maximum stimulation of these metallo-enzymes which differs from strain to strain [18]. It was reported that substrate hydrolysis by MBLs can be enhanced by the addition of zinc sulphate, and by further increasing the zinc sulphate concentration inhibition of these enzymes [19].

ZnO is a potential antibacterial agent due to its flexibility and broad spectrum of action against microbes [20,21]. Based on the electric charges stored in the Schottky barriers, it was recently revealed that nanostructured ZnO microparticles have outstanding antibacterial characteristics [20,22]. Many studies have linked the antibacterial activity of ZnO to the size of the component particles, with the nanometric size being more effective [23,24]. 

Here in the present work, we aimed to synthesize some novel 1,2,3-triazole-sulphadiazine-ZnO hybrids as potent antibacterial agents against multi-drug resistant strains, especially carbapenem-resistant ones. The newly designed hybrids were tested through docking, in vitro, and in vivo studies to prove the enzymatic inhibition, antibacterial activities, and the biodistribution and safety of the designed hybrids.

## 2. Results and Discussion

### 2.1. Chemistry

#### 2.1.1. Organic Synthetic Part

**Scheme 1:**
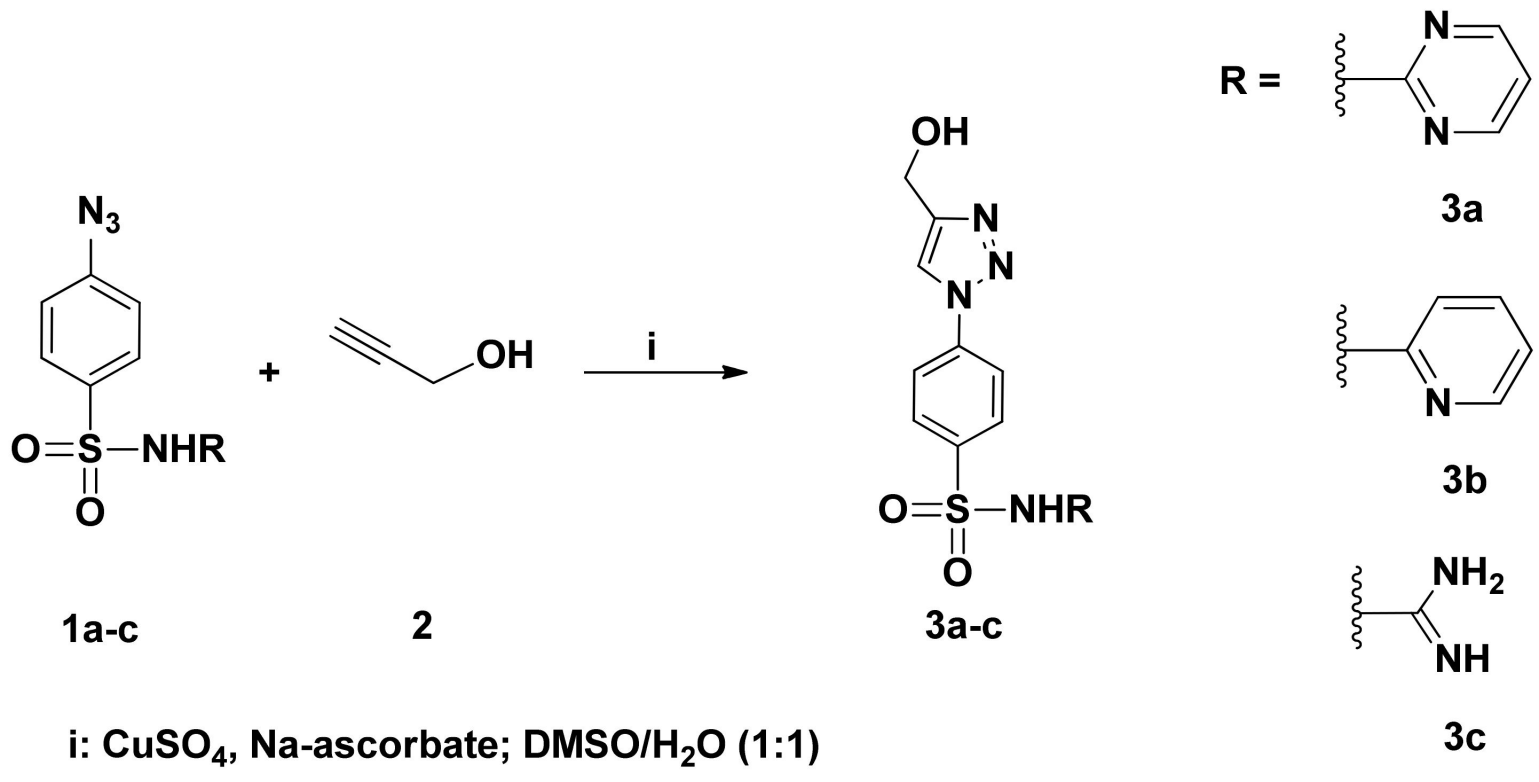
Click synthesis of 1,2,3-triazoles with sulfa-drug tethers **3a**–**c**.

The targeted 1,2,3-triazoles with sulfa-drug tethers were re-synthesized using our recently reported click procedure [25], as shown in Scheme 1. Click synthesis first required diazotization of sulfadiazine, sulfapyridine, and/or sulfaguanidine, followed by treatment with sodium azide to provide the corresponding sulfadiazine azides **1a**–**c**. Next, under optimized Cu(I)-assisted alkyne-azide cycloaddition conditions (CuSO_4_, Na-ascorbate; DMSO/H_2_O (1:1); 6–10 h room temperature), the freshly prepared sulfonamide azides were subsequently ligated to propargyl alcohol, yielding the desired 1,2,3-triazole-sulfonamide molecular conjugates **3a**–**c** in 86–90 percent yields.

All click adducts **3a**–**c** showed similar proton and carbon spectral data to those previously reported in our study [25]. The absence of the characteristic alkyne protons and carbons in their spectra and the appearance of diagnostic 1,2,3-triazolyl proton signals in the aromatic region confirmed their formation.

#### 2.1.2. Characterization of ZnO Nanoparticles

##### X-ray Diffractometer Analysis

**Figure 2:**
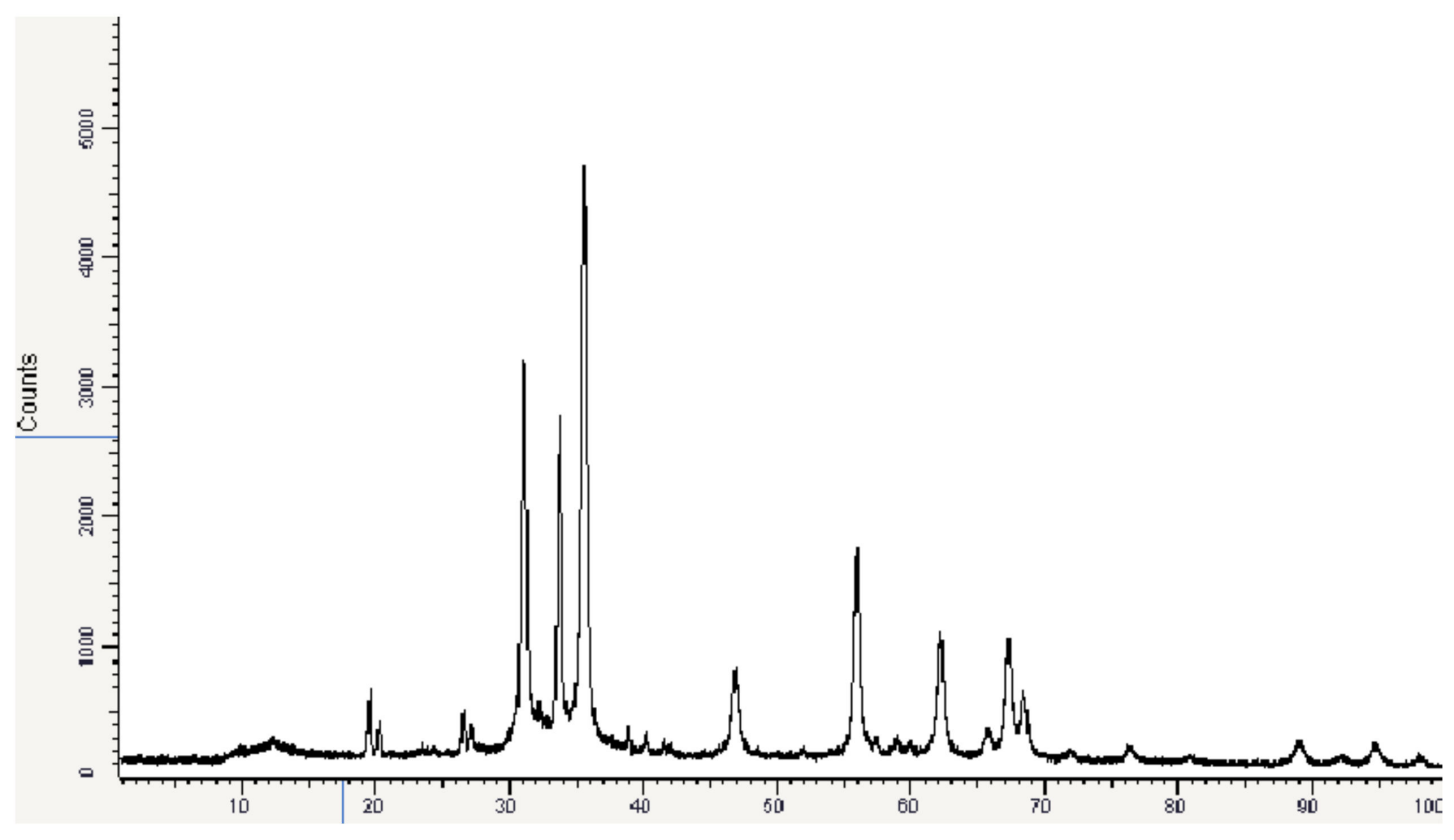
XRD pattern of ZnO NPs.

Only typical peaks for crystalline ZnO hexagonal phase were seen in the XRD pattern of ZnO NPs at (31.74o, 34.42o, 36.24o, 47.5o, 56.62o, 62.83o, 66.4o, 67.93o, 69.05o) planes (100), (002), (101), (102), (110), (103), (200), (112), and (201). The hexagonal ZnO wurtzite structure is clearly indexed by all the diffraction peaks (JCPDS no. 36–1451). The absence of diffraction peaks corresponding to impurities in the XRD patterns confirms the high purity of the produced products (Figure 2).

##### FT-IR Spectroscopy

**Figure 3:**
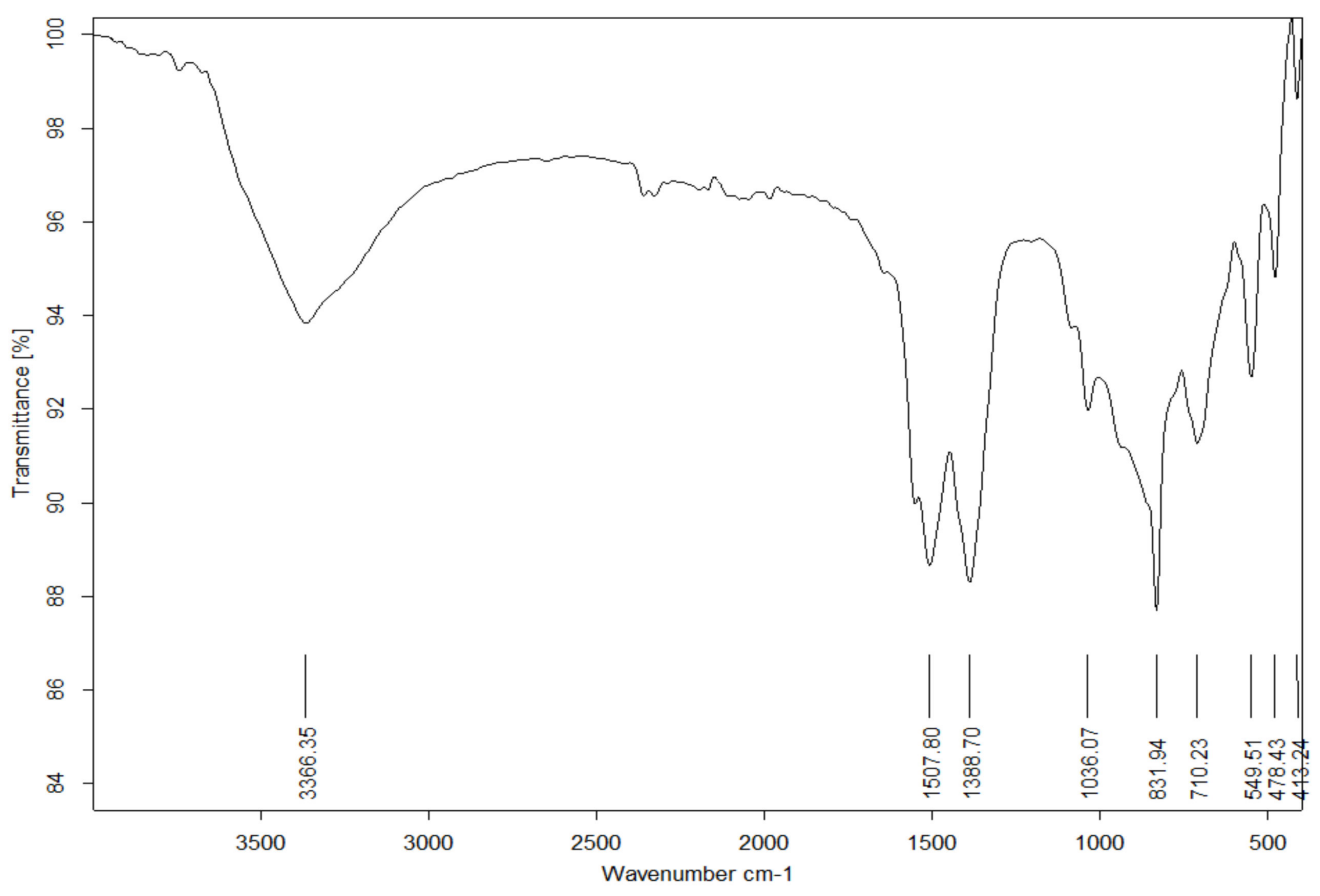
FTIR spectra of the ZnO NPs.

The FTIR spectra of ZnO nanoparticles are presented in Figure 3. Metal oxides generally give absorption bands in the fingerprint region, i.e., below 1000 cm^−1^ arising from inter-atomic vibrations. The absorption bands recorded at 1388.70 and 1507.80 cm^−1^ correspond to C=O and C-O bending vibrations, respectively. The O-H stretching, and deformation absorption bands observed at 3366.35 and 1036.07 cm^−1^, respectively, confirmed the adsorption of water on the metal surface.

The combination of the ZnO nanoparticles and the investigated sulfadiazine has been achieved by the mixing of 20 mg of ZnO and 20 mg of the synthesized sulfadiazines (**3a**–**c**).

#### 2.1.3. Molecular Modeling

Captopril is considered a potent MBLs inhibitor due to the presence of thiol and carboxylate functional groups which can coordinate with the zinc atoms in the enzyme active site. Several crystal structures of L-captopril and its stereoisomer D-captopril in the active sites of MBLs have been elucidated, yet there still remains uncertainty regarding how these inhibitors bind in the active site in solution [26]. While most studies have revealed that the thiol group of captopril binds to the zinc ions in the active site while other X-ray structures of the MBL–captopril complex show either no direct contacts between captopril and the zinc ions (e.g., with FEZ-1, B3 MBL) [27], or show the carboxylate group of captopril, rather than the thiol group, interacting with a single metal ion (e.g., with CphA, B2 MBL) [28].

**Figure 4:**
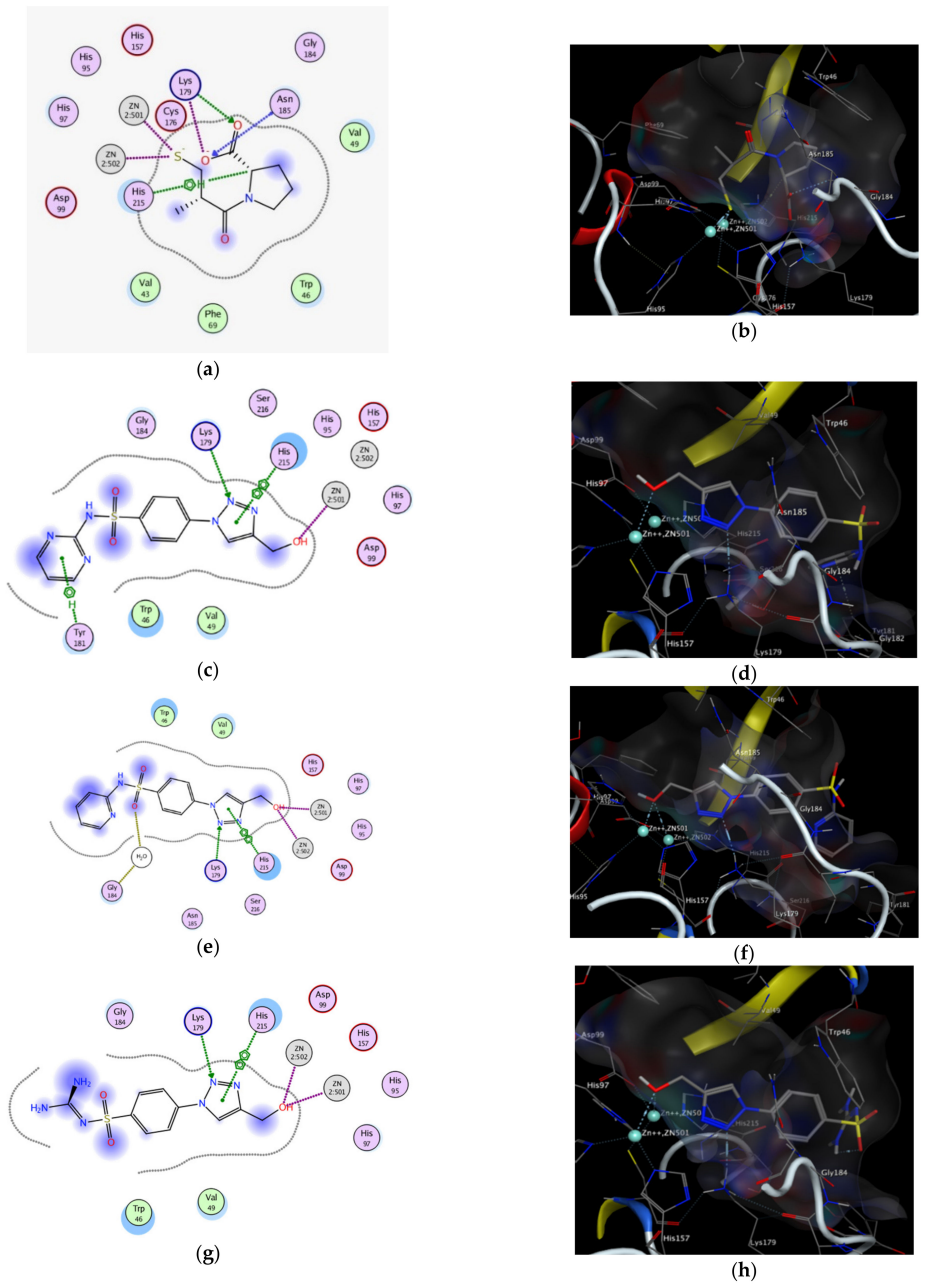
Docking study of the synthesized compounds against IMP-1. (**a**) Two-dimensional binding mode of L-captopril, (**b**) 3D binding mode of L-captopril, (**c**) 2D binding mode of compound **3a**, (**d**) 3D binding mode of compound **3a**, (**e**) 2D binding mode of compound **3b**, (**f**) 3D binding mode of compound **3b**, (**g**) 2D binding mode of compound **3c**, (**h**) 3D binding mode of compound **3c** inside IMP-1 active site.

**Figure 5:**
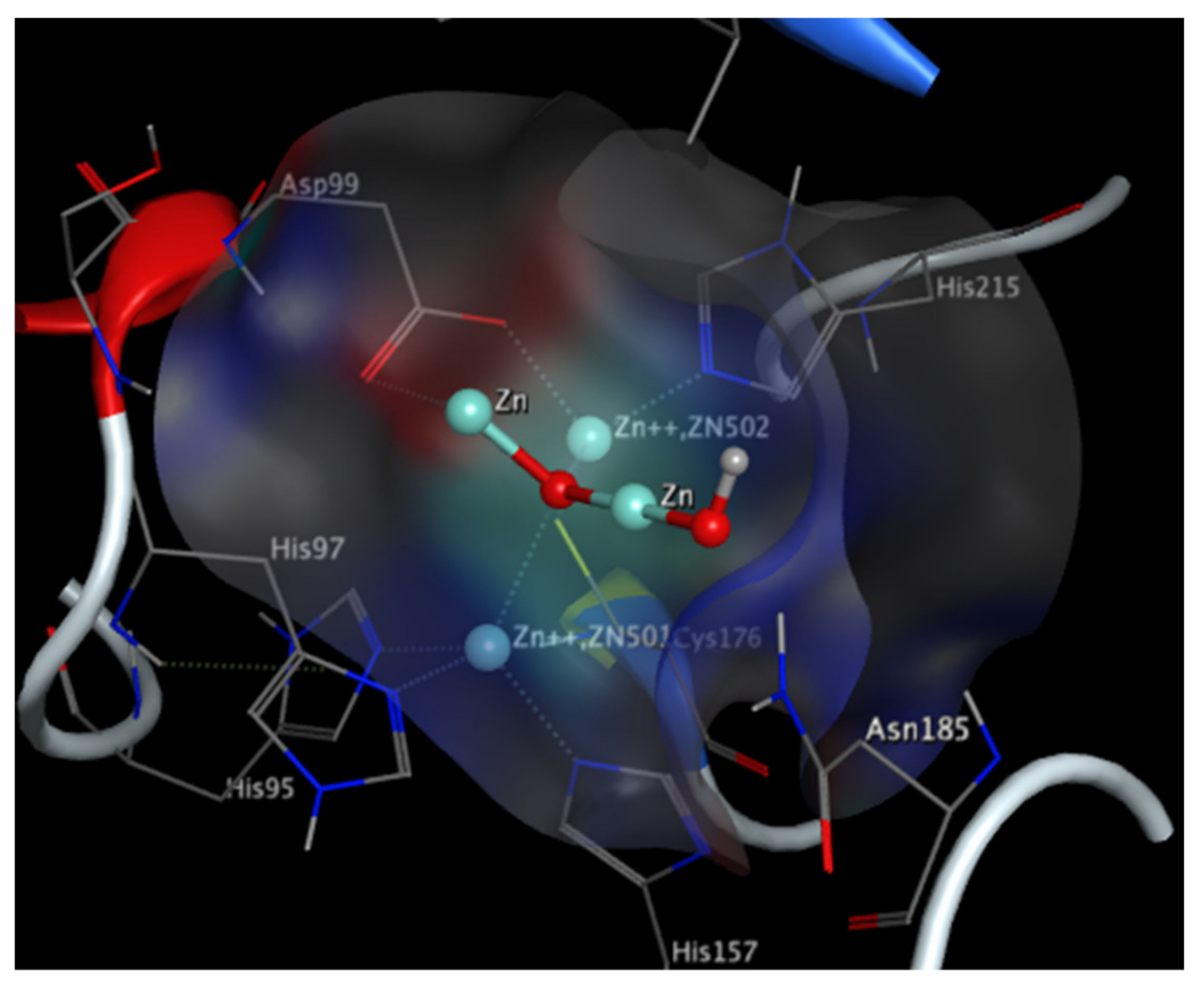
Three-dimensional binding mode of ZnO nanoparticles inside IMP-1 active site.

**Figure 6:**
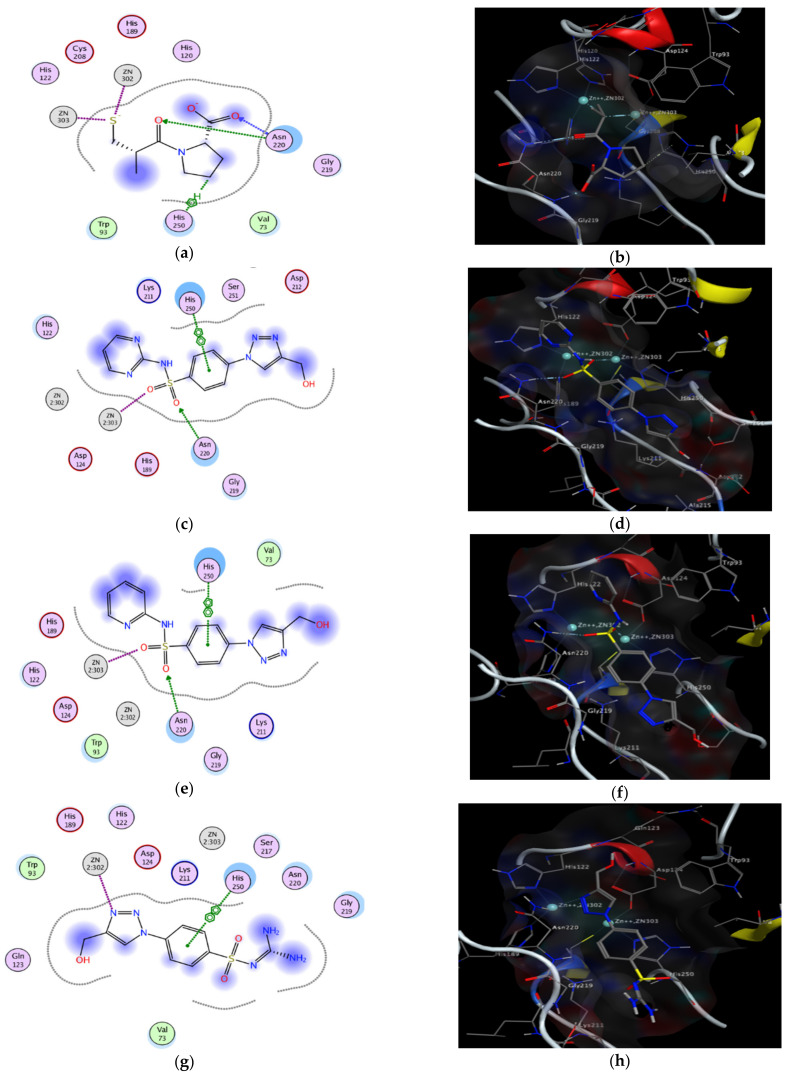
Docking study of the synthesized compounds against NDM-1. (**a**) Two-dimensional binding mode of L-captopril, (**b**) 3D binding mode of L-captopril, (**c**) 2D binding mode of compound **3a**, (**d**) 3D binding mode of compound **3a**, (**e**) 2D binding mode of compound **3b**, (**f**) 3D binding mode of compound **3b**, (**g**) 2D binding mode of compound **3c**, (**h**) 3D binding mode of compound **3c** inside NDM-1 active site.

In the current study, molecular docking was performed to predict the binding scores in addition to the binding mode of the tested compounds in addition to ZnO nanoparticles within the active site of the target. The docking score could compute the binding energy to select orientations with the lowest energy state. It also could be used as an indicator of the relative strength of interactions with the active site. The predicted docking scores and binding features of the investigated compounds towards the enzyme targets were listed in Table 1 and Table 2. In addition, the 2D and 3D representations of interactions of the examined compounds with the key amino acid residues of these enzymes are illustrated in Figure 4, Figure 5 and Figure 6.

##### Imipenemase-1 (IMP-1) Enzyme

The IMP-1 and L-captopril complex showed the thiol group interacting with the metal ions in the active site which displaced a bridging water molecule of the enzyme (essential for the enzyme activity). H-bond interactions between the captopril carboxylate and the Lys179 as well as Asn185 were also observed in addition to hydrophobic interaction between the pyrrolidine ring His215 (Table 1). Docking results indicated that all tested compounds possessed remarkable binding scores higher than the co-crystallized ligand L-captopril, in addition to a similar binding mode to that exhibited by the L-captopril indicating a valid reliable docking approach. It was also found that the tested compounds displayed a strong pattern of interactions within the IMP-1 active site including hydrogen bonds, hydrophobic interactions, as well as coordination with zinc atoms (Figure 4). The docking study of ZnO nanoparticles showed that the oxygen atom of ZnO can coordinate with the two catalytically required zinc ions at the enzyme active site, displacing the nucleophilic water molecule, leading to enzyme inhibition (Figure 5). It is worth mentioning that the results of the practical IMP-1 inhibition screening were compatible with the theoretical docking results of the examined compounds.

##### New Delhi Metallo-β-Lactamase (NDM-1) Enzyme

**Figure 7:**
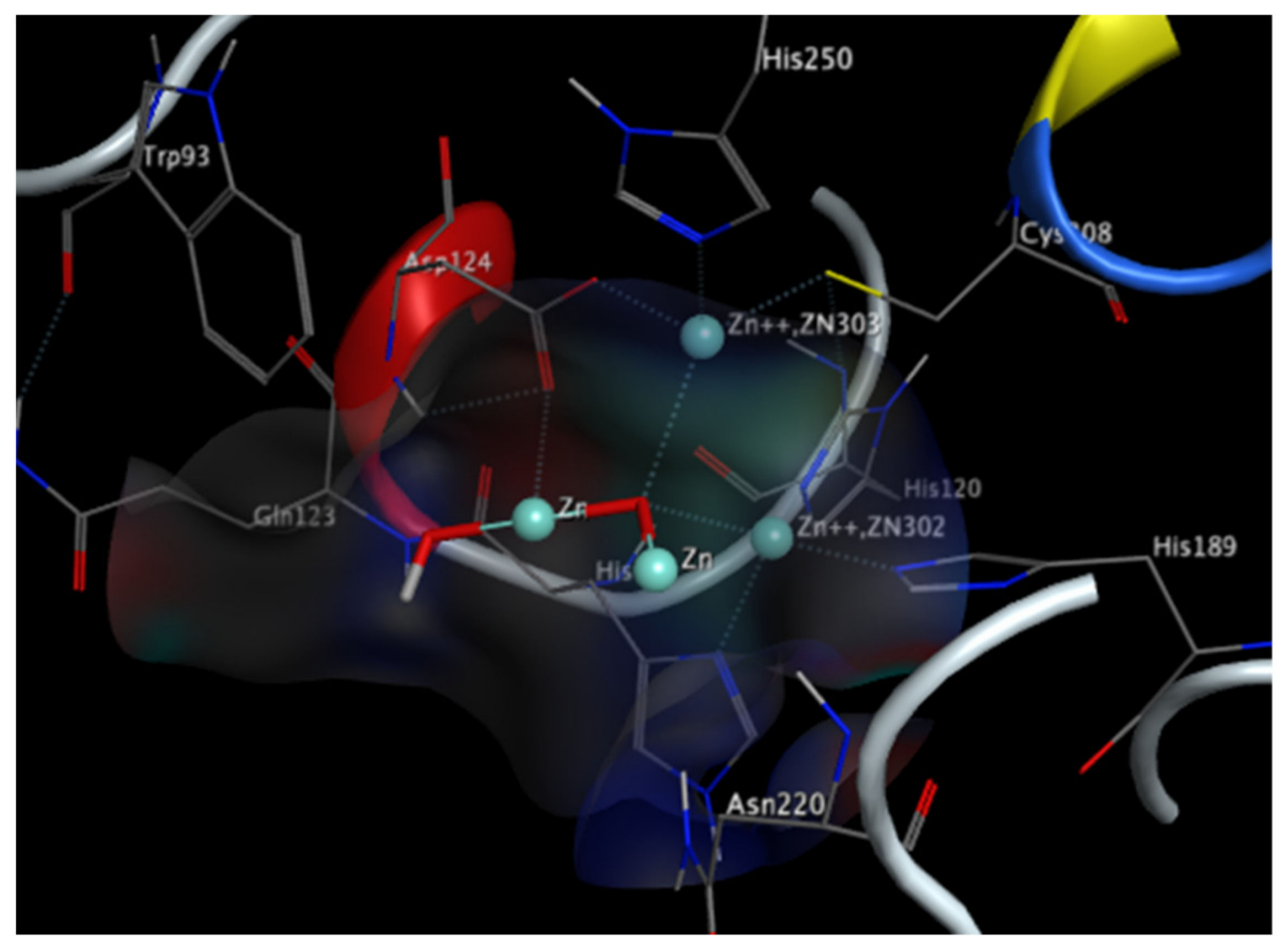
Three-dimensional binding mode of ZnO nanoparticles inside NDM-1 active site.

The three-dimensional model of NDM-1 complexed with captopril declared that the thiol group coordinated with zinc atoms and displaced the nucleophilic water molecule, leading to a competitively inhibited enzyme. It also displayed a hydrophobic interaction with His250 in addition to a hydrogen bond with Asn220 (Table 2). Results of the docking studies revealed that test compounds **3a**–**c** showed promising docking scores comparable with the co-crystallized ligand L-captopril. Amazingly, compound **3a** showed the best scoring results higher than L-captopril (Figure 6). These compounds formed many types of interactions such as coordination with zinc atoms at the active site of NDM-1, hydrogen bonds in addition to hydrophobic interactions, and are considered to be promising inhibitors. Interestingly, it was observed that ZnO nanoparticles possessed a high affinity toward NDM-1 due to the coordination of the ZnO oxygen atom with the two essential zinc ions at the enzyme active site (Figure 7).

### 2.2. In Vitro Analyses

#### 2.2.1. Detection of the IPM and NDM Genes Presence in the Bacterial Isolates

**Figure 8:**
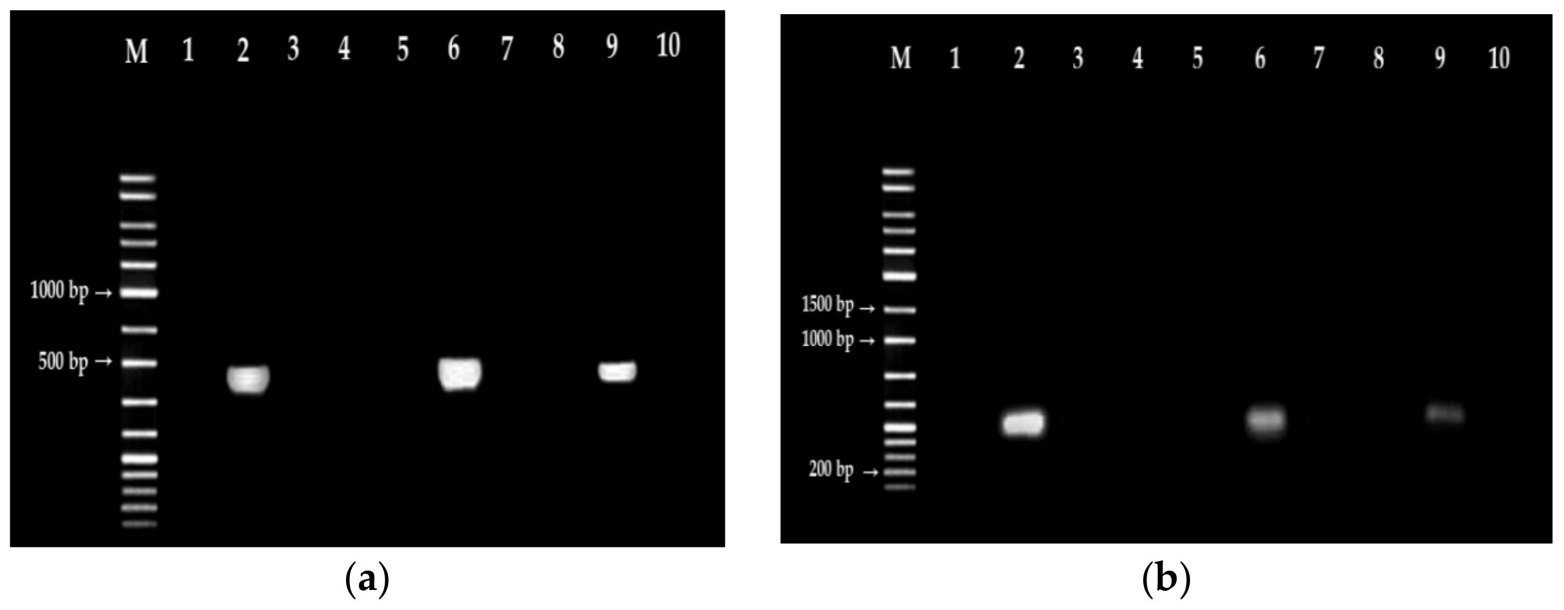
Agarose gel electrophoresis showing: PCR detection for NDM (**a**) and IPM (**b**) genes. *K. pneumoniae* ATCC 13883 in lane 1 while *K. pneumoniae* tested strains were in lane from 2 to 10.

PCR was applied to the *K. pneumoniae* (Kp) strains numbered from 1 to 9 in comparison to *K. pneumoniae* ATCC 13883 (Figure 8). Data revealed that NDM and IPM genes were present in 30% (*K. pneumoniae* strains 1, 5, and 8) of all the tested *K. pneumoniae* strains (Table 3). Strain numbers 1, 5, and 8 were selected for further analyses. 

#### 2.2.2. MBLs Inhibition Activity against IMP-1 and NDM-1

The inhibitory effects of the synthesized compounds **3a**–**c** and their hybrids were tested against IMP-1 and NDM-1. Tested compounds were tested for further kinetic studies to determine their inhibition constants (Ki values) and modes of inhibition whether competitive or uncompetitive inhibition. The tested compounds inhibited the enzymes both competitively and noncompetitively (Table 4). This mixed inhibition mode suggested that these inhibitors could bind to the active site of MBLs (competitive inhibition) as well as form a ternary enzyme–substrate–inhibitor (ESI) complex that inhibited substrate hydrolysis (uncompetitive inhibition) [6]. These compounds demonstrated promising inhibition constants in the low micromolar range towards NDM-1 (8.5–14.7 µM) in the competitive mode, as well as 10.9–17.8 µM and 5.1–9.4 µM against IMP-1 in the competitive and noncompetitive modes, respectively.

#### 2.2.3. Antibacterial Activity against Metallo-β-Lactamase Producing *K. pneumoniae*

**Figure 9:**
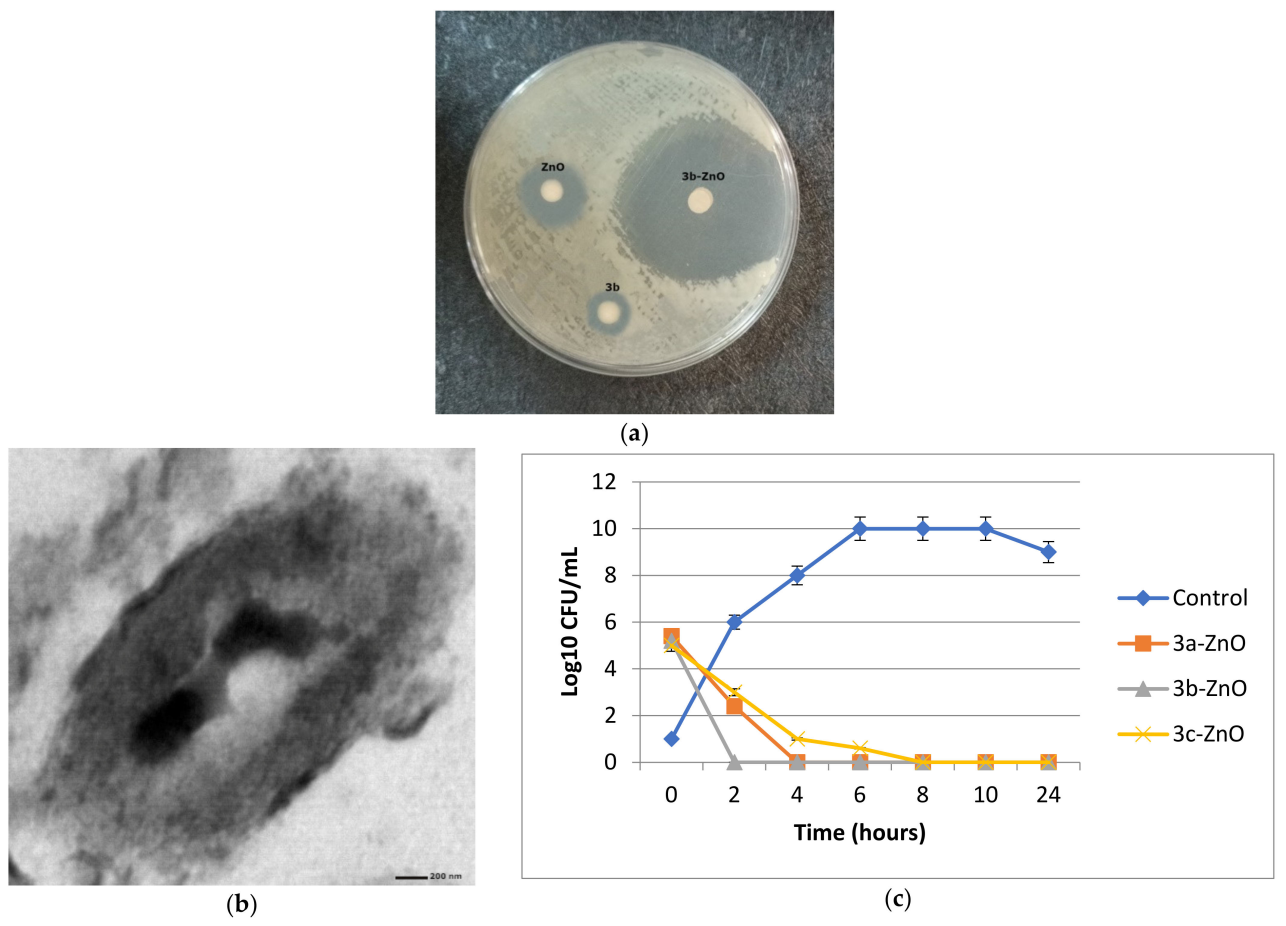
Antibacterial effect of the synthesized compounds and their hybrids against Kp5. (**a**) Disc diffusion method, (**b**) transmission electron microscopic study of Kp5 treated cell, and (**c**) time-kill curve.

The antimicrobial activity of the synthesized compounds **3a**–**c** and their hybrids was assessed against resistant clinical isolates of *K. pneumoniae* (Kp1, Kp5, and Kp8). Remarkable synergistic activity was shown between the synthesized 1,2,3-triazole-sulphadiazine (**3a**, **3b**, and **3c**) and ZnO nanoparticles. Compound **3b**-ZnO showed the highest antibacterial activity against the tested metallo-β-lactamase producers (inhibition zone (IZ) diameter reached 43, 35, and 40 mm while the minimum inhibitory concentration (MIC) reached 2, 4, and 2 µg/mL against Kp1, Kp5, and Kp8, respectively) (Table 5). Kp5 was the most resistant strain and hence was selected for further analyses. Further investigation through a time-kill curve proved the superior activity of **3b**-ZnO with noticed eradication of the bacterial growth after only four hours. Moreover, a transmission electron microscope study of the bacterial treated cells with **3b**-ZnO proved the cell wall disruption and cellular components leakage (Figure 9). 

The significance of ZnO nanomaterial characteristics in antibacterial activity, on the other hand, is still little known. The formation of reactive oxygen species (ROS) such as free radicals, oxygen ions, and peroxides, e.g., oxygen (O_2_), hydrogen peroxide (H_2_O_2_), hydroxyl ion (OH), hydroxyl radicals (OH^•^), peroxide (O_2_^−2•^), and anion superoxide (O^2‒•^), is one of the primary contributors in the ZnO action mechanism [29]. The penetration of Zn^2+^ cations into bacterial cells is aided by the fact that the bacterial membrane has a negative charge, resulting in electrostatic attraction. The poisonous Zn^2+^ cation has the following effects on the microorganism: (i) instability of the membrane and increased permeability due to direct contact with bacterial membranes [30], (ii) penetration and reaction with nucleic acids, resulting in the inactivation of respiratory system enzymes [30], and (iii) release of additional Zn^2+^ ions into the cell, which can trigger cytokine production and cytotoxicity [31]. The basic function of ROS is to create oxidative stress in cells; however, a large amount of negatively charged ROS on the microorganisms’ outer surface can counteract the Zn^2+^ cations’ efficiency. Furthermore, when NP-ZnO comes into direct touch with bacteria, its surface imperfections might produce abrasions [20,31].

#### 2.2.4. Cytotoxicity Study

**Figure 10:**
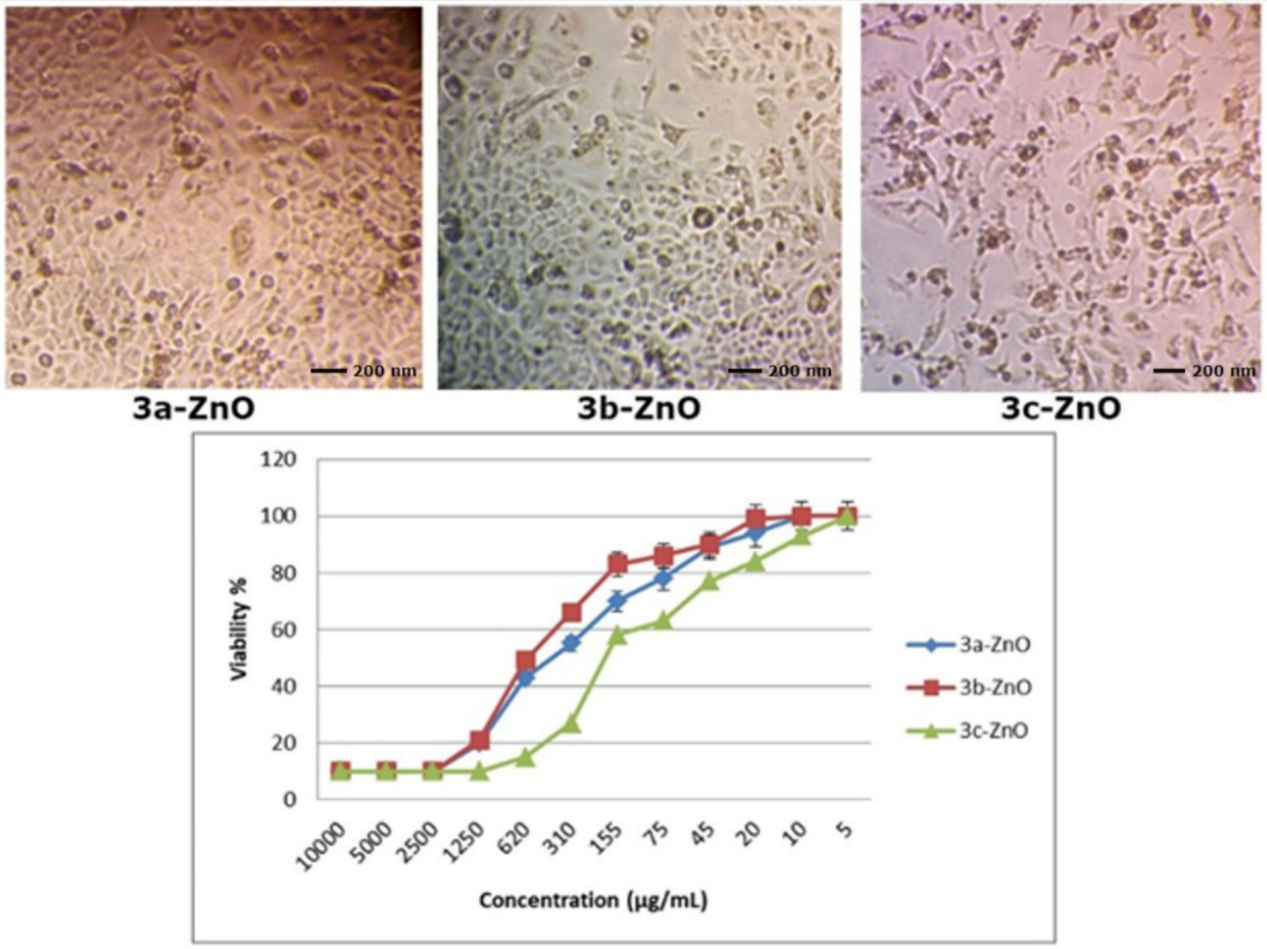
Cytotoxic effect of the synthesized hybrids.

1,2,3-triazole-sulphadiazine-ZnO hybrids namely **3a**-ZnO, **3b**-ZnO, and **3c**-ZnO were tested for their in vitro cytotoxicity in a normal lung cell line (BEAS-2Bs cell line). Higher cell viability was observed with **3b**-ZnO. Compound **3b**-ZnO had an IC50 of 620 g/mL, while compounds **3a**-ZnO and **3c**-ZnO had IC50s of 450 and 160 g/mL, respectively (Figure 10). The observed data proved the potent safety and the possible biomedical applications of the prepared1,2,3-triazole-sulphadiazine-ZnO hybrids, especially **3b**-ZnO.

### 2.3. In Vivo Studies

#### 2.3.1. Biodistribution of the 1,2,3-Triazole-sulphadiazine-ZnO Hybrids in Lungs of Uninfected Rats

**Figure 11:**
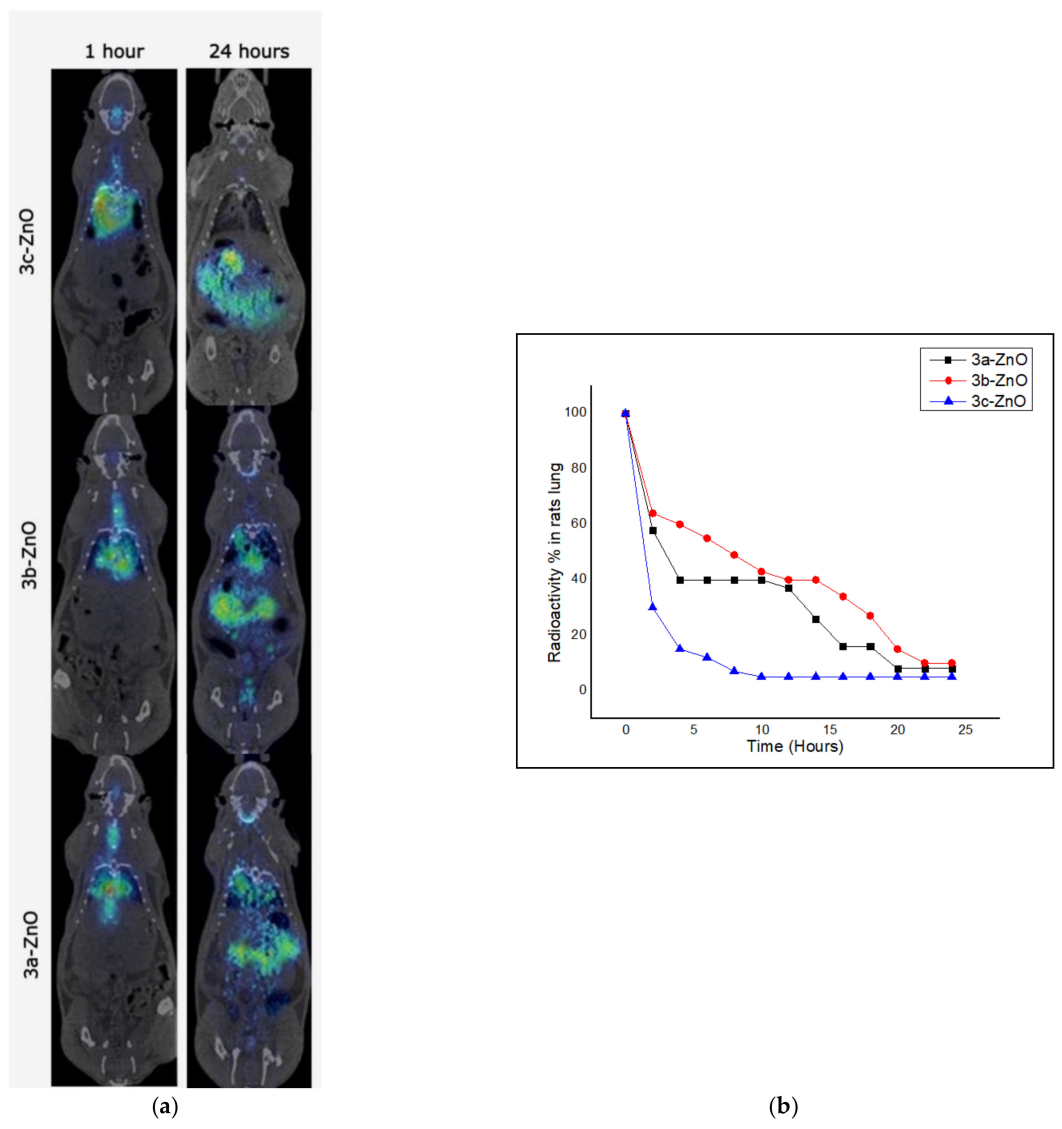
Endotracheal aerosolization in uninfected rats through PET-CT images of the biodistribution of radiolabeled compounds (**a**). Concentration of the radiolabeled compounds in the uninfected rats’ lungs versus time (**b**).

Positron emission tomography-computed tomography (PET-CT) images for [^124I^]**3c**-ZnO, [^124I^]**3a**-ZnO, and [^124I^]**3b**-ZnO were acquired in groups of five normal (uninfected) rats. When [^124I^]**3c**-ZnO was endotracheally aerosolized, it was quickly removed from the lungs, within 24 h, and there was no signal of [^124I^]**3c**-ZnO residues in the rats’ lungs (Figure 11a). On the contrary, following administration, a considerable proportion of both [^124I^]**3a**-ZnO and [^124I^]**3b**-ZnO hybrids remained detectable within the rats’ lungs after 24 h of endotracheal aerosolization administration of the labeled hybrids.

#### 2.3.2. Residence Time of the 1,2,3-Triazole-sulphadiazine-ZnO Hybrids in Lungs of Uninfected Rats

The tendencies seen in the above-mentioned observations were verified when the radioactivity in the lungs was quantified. To calculate the biological half-life (t_1/2_) of the radiolabeled substances in the rats’ lungs, the exact concentration of radioactivity in the rats’ lungs at different time intervals was plotted in a monoexponentially decay equation. The residence duration in the lungs of [^124I^] **3a**-ZnO (t_1/2_ 2.18 h) was 23.7 percent longer, compared to when administered as [^124I^] **3b**-ZnO (t_1/2_ 4.91 h), it was 85.3 percent longer. On the other hand, [^124I^] **3c**-ZnO had a relatively low residence period in the lungs (t_1/2_ 0.13 h) (Figure 11b).

#### 2.3.3. Bacterial Load Assessment

**Figure 12:**
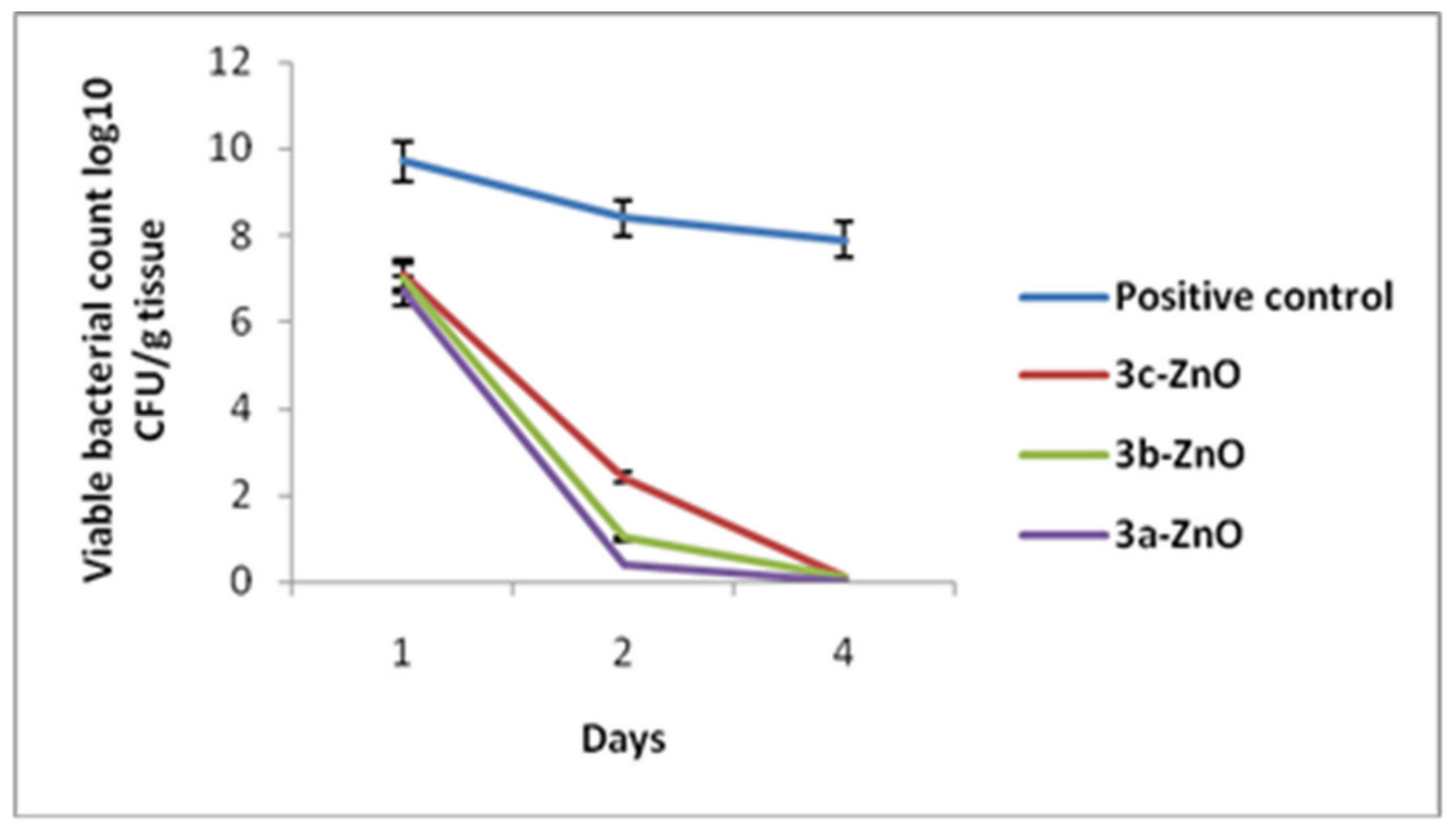
Bacterial load assessment in the lungs of pneumonic rats.

At the beginning of the experiment, the rats received intranasal inoculation with 50 μL bacterial suspensions 10^7^CFU/mL of *Klebsiella pneumoniae* strain 5 (Kp5). At a specific time, interval, the viable bacterial cells were counted (CFU/g tissue). Data in Figure 12 showed a significant reduction in the Kp5 viable cell count among all the tested treatments in relation to the control group. However, there was no significant difference in the viable bacterial count between **3a**-ZnO and **3b**-ZnO treatments (Figure 12).

#### 2.3.4. Histopathological Investigations

##### Light Microscopic Investigations

(a) First Interval

**Figure 13:**
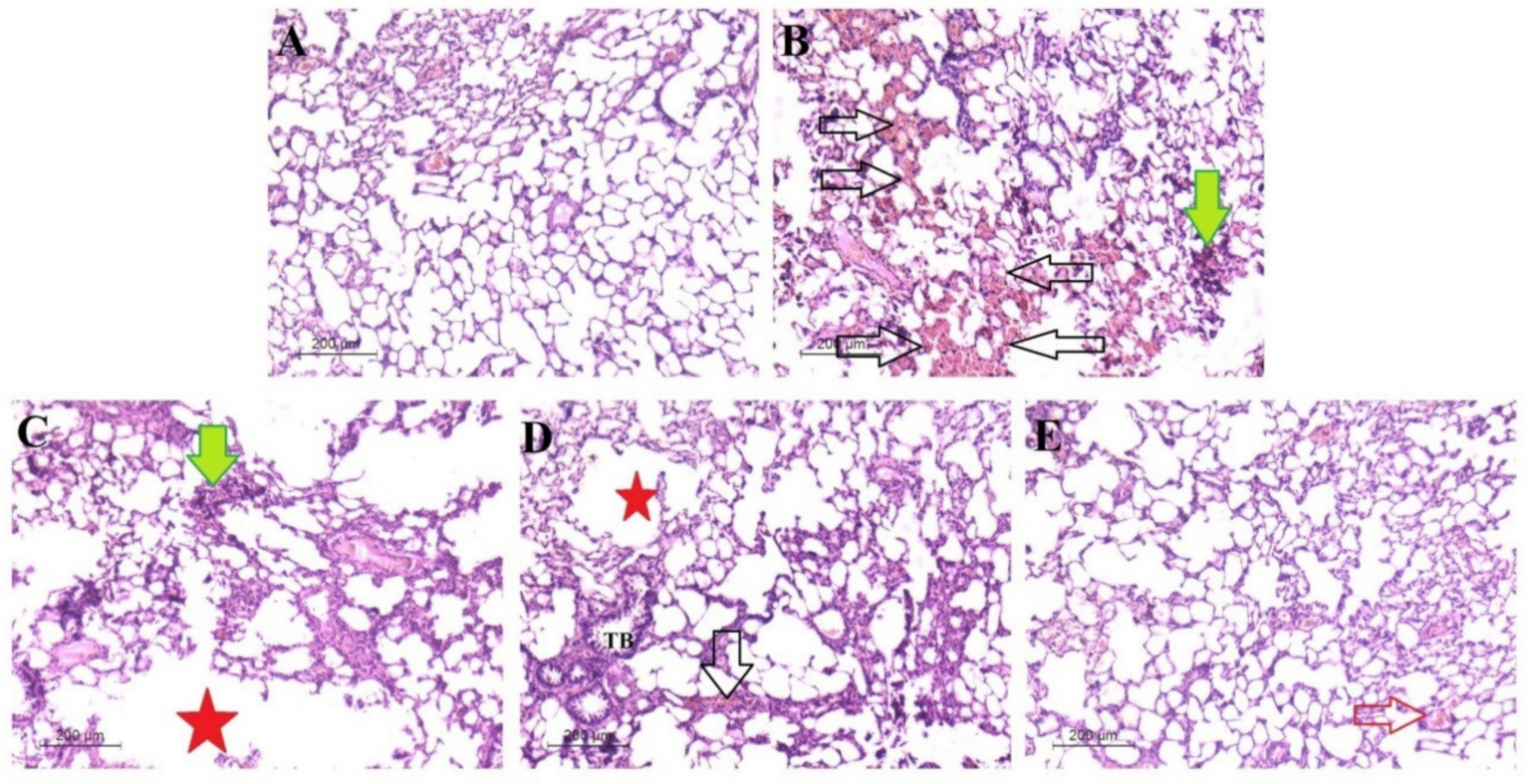
Photomicrograph of rat lung infected with Kp5 and given endotracheal aerosolization various preparations of 1,2,3-triazole-sulphadiazine-ZnO hybrids as treatments, 48 h post-infection. (**A**) Negative control group. (**B**) Positive control; (**C**) **3c**-ZnO; (**D**) **3a**-ZnO; and (**E**) **3b**-ZnO. In which black arrows refer to focal accumulations of macrophages (in **B**); red arrows refer to normal small blood vessels for gas exchange; red stars (alveolar histiocytosis); green arrows refer to densely packed cluster of lymphocytes associated lymphoid tissue; TB refers to terminal bronchioles found to be alienated with normal histological structure in (**D**) and damaged ones observed in (**C**). H&E stain (magnification: 200×).

Previous histological studies revealed significant changes in the inflammatory cell response depending on the therapy type. We followed up on these experiments by comparing histological samples from both intervals (48 and 96 h post-infection). After 48 h after pneumonia induction, the variations in the pulmonary epithelial cell responses in the **3b**-ZnO-treated group were striking, showing the bacteria’s treatment management. Although the **3a**-ZnO-treated lungs showed significant epithelial cell hypertrophy as compared to the control sample, alveolar histiocytosis is minimal, with typical terminal bronchioles. Interestingly, histological inspection of the negative control and **3c**-ZnO-treated lungs reveals substantial regions of lymphocyte infiltration that were not present in the other two treated lungs (Figure 13). Higher magnification was added as Supplementary Figure S3.

(b) Second Interval

**Figure 14:**
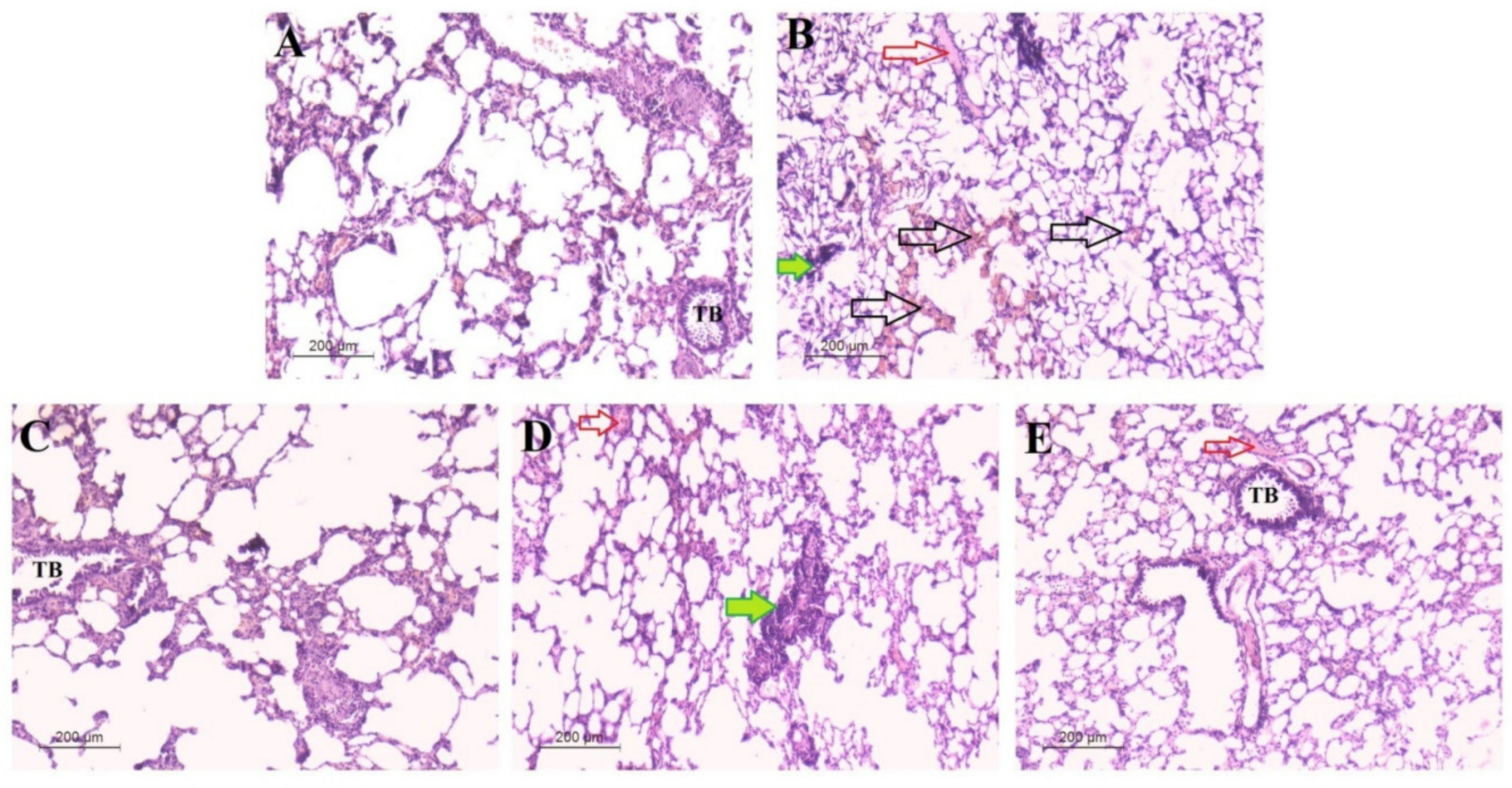
Photomicrograph of rat lung infected withKp5 and given endotracheal aerosolization various preparations of 1,2,3-triazole-sulphadiazine-ZnO hybrids as treatments, 96 h post-infection. (**A**) Negative control group. (**B**) Positive control; (**C**) **3c**-ZnO; (**D**) **3a**-ZnO; and (**E**) **3b**-ZnO. Black arrows refer to increased macrophage focal accumulations in (**B**), with some remnants of macrophage accumulation only observed in (**D**); red arrows refer to the appearance of normal small blood vessels for gas exchange; green arrows refer to lymphatic-associated lymphoid tissue in (**B**,**C**) that is not symptoms of alveolar histiocytosis found in (**C**,**D**). Fewer signs of histiocytosis persisted in (**E**) with its elimination in (**A**,**B**). H&E stain (magnification: 200×).

In the current study, histopathological lesions in rats’ lungs were compared to normal control rats’ lungs. The principal results showed discrete regions of necrosis and mild to moderate neutrophilic infiltration with erythema and edema in the lungs of non-treated control (Figure 14) and 1,2,3-triazole-sulphadiazine-ZnO hybrids-treated rats collected after 96 h post-infection. Perivascular and peribranchial lymphocytic infiltration was limited in the different treatment groups. This demonstrated the treatment’s capacity to control the infection’s excessive state of inflammation. In contrast, extensive interstitial infiltration caused alveolar histiocytosis in the **3c**-ZnO-treated group reflecting the non-significant effect of this treatment. Higher magnification was added as Supplementary Figure S4.

##### Transmission Electron Microscopical Study

**Figure 15:**
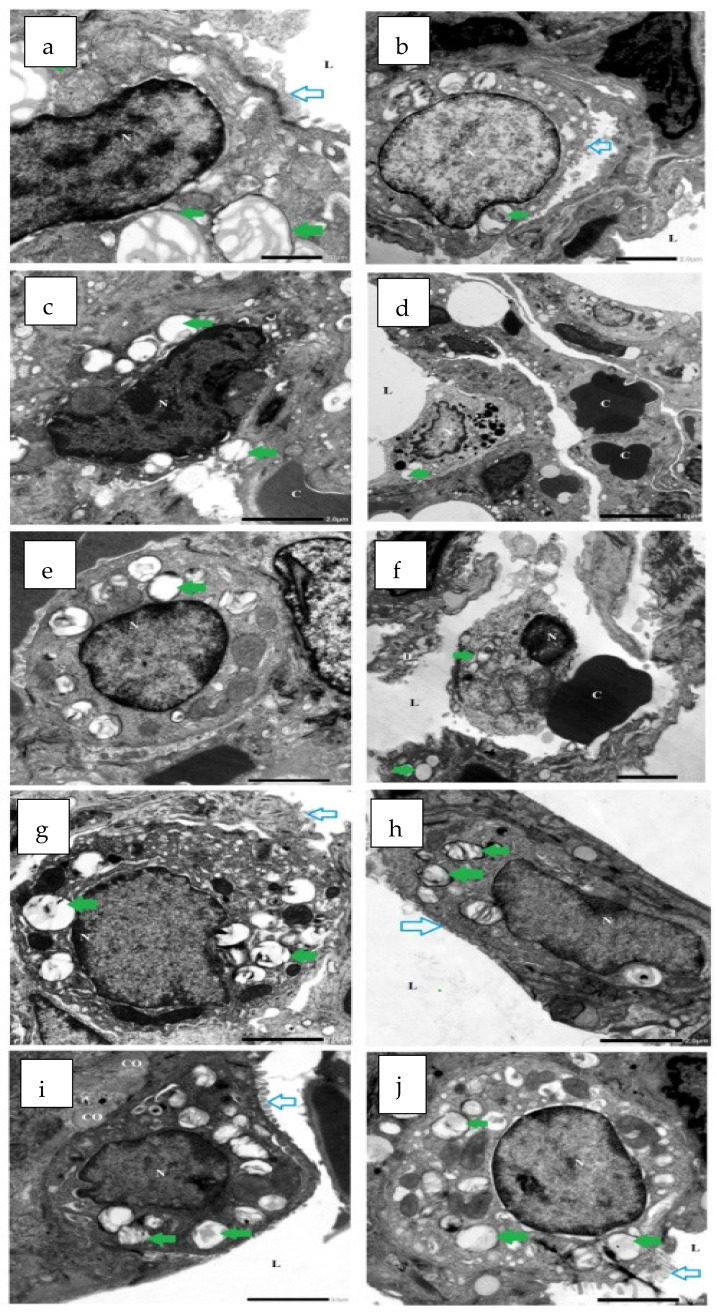
Transmission electron microscope study of the first interval (**a**,**c**,**e**,**g**,**i**) and the second interval (**b**,**d**,**f**,**h**,**j**) of the experimental rat groups. Note: (**a**,**b**) and b represent the control group, (**c**,**d**) represent the positive control group, (**e**,**f**) represent group 3, (**g**,**h**) represent group 4, and finally (**i**,**j**) represent group 5. Nucleus (N); alveolar lumen (L); cellular debris (D); congestion (C); luminal micro villi (blue arrows); lamellar bodies (green arrows).

Transmission electron microscopy was utilized to analyze the ultrastructural alterations in lung tissues at two time intervals to track the histopathological changes. The first interval revealed distinct cell borders, nuclear membranes, and osmiophilic lamellar bodies. Pneumocyte type II, which has typical lamellar bodies and big vesicular nuclei with short luminal microvilli, were observed as forms of alveolar cell continuous with an alveolar lumen. Similar findings were observed in the **3b**-ZnO-treated group, which displayed well-organized epithelial microvilli and a large number of lamellar bodies. The architecture of the **3a**-ZnO-treated group was more or less typical, with a low number of microvilli. Furthermore, numerous lamellar bodies were seen to be devoid of lamellation. The positive control group demonstrated a disordered nuclear membrane with lamellar dissolution in lamellar bodies and septal capillary congestion. The **3c**-ZnO-treated group outperformed the positive control group in terms of a smooth nuclear membrane appearance. This group still has septal capillary congestion (Figure 15).

The second interval was also briefly highlighted by displaying the distinction from the preceding one. As mentioned above, the control group has normal type II alveolar cells in constituent with the typical appearance in the **3b**-ZnO-treated group, as well as the appearance of normal lumen, emphasizing the importance of type II cells in reducing breathing. In this time interval, the **3a**-ZnO-treated group had more lamellae and more structured microvilli.

The positive control group had the worst outcomes due to an increase in the number of clogged septal capillaries, which plays a major role in diminishing the alveolar lumen and hence the efficiency of breathing. The strategy of improved outcomes in the first interval concerning the **3c**-ZnO-treated group no longer lasts, although the results appear to be similar to the positive control after a short period of time.

## 3. Materials and Methods

### 3.1. Chemistry

#### 3.1.1. Synthesis of 1,2,3-Triazoles Bearing Sulfa-Drug 3a−c

The click products **3a**–**c** were prepared and resynthesized in accordance with our previous work [25]. Detailed synthesis steps and compounds characterizations were added as Supplementary Materials (Figures S1 and S2)

#### 3.1.2. Synthesis of ZnO Nanoparticles

Precursor zinc acetate dihydrate (0.1 M) was refluxed in diethylene glycol and triethylene glycol at 180 °C and 220 °C, respectively, to produce ZnO nanoparticles. The reaction stirred for 3 h in the presence of sodium acetate (0.01 M). The solution was maintained on a magnetic stirrer at 80 °C for 1.5 h prior to refluxing. Following the end of the reflux action, the samples were centrifuged at 8000 rpm for 15 min and rinsed three times with distilled water and ethanol. It was then dried overnight at 80 °C.

#### 3.1.3. Combination between 1,2,3-Triazole-sulphadiazine and ZnO NPs

The combination of the ZnO nanoparticles and the investigated sulfadiazines in order to maximize the potential biological activity has been achieved by the mixing of 20 mg of ZnO and 20 mg of each synthesized sulfadiazine (**3a**–**c**) one at a time.

#### 3.1.4. Molecular Modeling

Docking studies were carried out using the Molecular Operating Environment software (MOE) to predict the binding affinity and binding interactions of the investigated compounds **3a**–**c** as well as ZnO nanoparticles with the target MBLs enzymes (IMP-1 and NDM-1) in order to develop novel potential inhibitors for these main targets [12].

##### Bacterial Enzymes Preparation

For molecular docking calculations, the X-ray crystal structures of the protein targets in complex with co-crystallized ligand L-captopril; IMP-1 (PDB ID: 4C1F) [32] and NDM-1 (PDB ID: 4EXS) [33] were obtained from the Protein Data Bank (PDB) website and were taken as templates, followed by the addition of all missing hydrogen atoms. For molecular docking calculations, the target proteins were prepared by employing the default “Structure preparation” module settings. Then, ‘Site Finder’ feature of MOE was employed to detect the receptor binding site.

##### Database Generation and Optimization

Three tested compounds were drawn using the ChemDraw program in addition to the hexagonal ZnO wurtzite structure (ID: mp-2133) obtained from the materials project website and collected in the database in MOE software. This database was subjected to displaying hydrogens, default energy minimization as well as computation of partial charges. A triangular matcher algorithm was applied to set the ligand placement. The default scoring function was alpha HB which generated the top 5 non-redundant poses of the lowest binding energy conformers of the tested compounds. Docking was conducted for the prepared database with induced fitting protocol to record the best possible molecular interactions. The consensus scoring in Kcal-mol was calculated using two scoring functions, alpha hydrogen bonding and London Dg forces. Results were listed based on the S-scores with RMSD values <2 Å. Molecular docking results are often validated using a training set of experimental ligand–protein complexes, and the accuracy of this docking software is mainly dependent on the used training set. It is important to ensure that the used program can replicate the binding mode of a known reference inhibitor (L-captopril) for the studied enzyme. Finally, conformers that give the best docking scores and ligand–enzyme interactions were selected and analyzed.

### 3.2. Biological Evaluation

#### 3.2.1. Microorganisms

Multi-drug resistant strains of *Klebsiella pneumoniae* under test were kindly identified and provided by the Microbiology Department’s Strain Bank, at the main University Hospital, Alexandria, Egypt.

#### 3.2.2. In Vitro Analyses

##### Determination of the Metallo-β-Lactamases Production Using Molecular Technique

Bacterial suspensions were freshly prepared, and the cells were harvested after 18 h incubation at 35 ± 2 °C. Pellets of ≈2 × 109 CFU/Ml were processed for DNA extraction using the GeneJET Genomic DNA Purification Kit. IPM and NDM primers were designed where IPM F primer: AC(G/A)GG(C/G/T)GGAATAGAGTGGCTTAA(T/C)TCTC and the R primer: TTCAGG(C/T)A(A/G)CCAAACYACTASGTTATCT while NDM F primer: CGAAAGTCAGGCTGTGTTGCGC and R primer: GACCGCCCAGATCCTCAACTG. The final concentration of working primers amounted to 1 pmol. The final concentration of dNTPs was typically 2 Μm for each deoxynucleotide. The initial denaturation step was set for 4 min at 95 °C. Annealing temperature used was (50, 53, 55, and 58 °C one at a time) for 60 s. The extension temperature was 72 °C for 1 min. The number of cycles was 35. A final extension step for 10 min was permitted at 72 °C.

##### In Vitro MBLs Inhibition Activity against IMP-1 and NDM-1

Assays of enzyme inhibition were measured in the absence and presence of 5 M of compound **4a**–**c** and their ZnO conjugated hybrids to determine their inhibitory activity (IMP1 and NDM-1). CENTA (70 µM), a chromogenic cephalosporin substrate, was employed [34,35]. The chromophore 4-nitrophenolate is formed when MBLs hydrolyze CENTA, and its generation may be detected spectrophotometrically using a plate reader at = 405 nm (at pH 7.0, ε = 6400 M^−1^ cm^−1^). Assays of kinetics of the inhibition experiments were carried out in accordance with earlier research. Non-linear regression was used to examine the inhibition data [36].

##### Antibacterial Activity of the Prepared Compounds

The antibacterial activities of the prepared compounds were evaluated against different β-lactam-resistant *Klebsiella pneumoniae* strains. Disc-diffusion method, minimum inhibitory concentration (MIC), bacterial lethality curves, and transmission electron microscopic study were used to assess the potential antibacterial activity of the prepared compounds according to Elnaggar [37].

##### Cytotoxicity Study in Lung (BEAS-2Bs) Cell Line

Cytotoxicity test was determined in a lung cell line (BEAS-2Bs). Cells were grown in DMEM medium supplemented with 10% FBS, 100 units/mL of penicillin, and 100 mg/mL of streptomycin. Cultures were maintained in a humidified atmosphere with 5% CO_2_ at 37 °C. Each sample was diluted in DMEM complete media at 37 °C to give a stock solution. Ten concentrations of two-fold serial dilutions were performed according to the client’s request. Briefly, confluent monolayers of lung cells were grown in 96-well microtiter plates for 24 h. Cells were incubated with various concentrations of the test compounds in triplicate at 37 °C in a CO_2_ environment for 72 h. After that, 20 μL of 5 mg/mL MTT was added to each well and incubated at 37 °C for 4 hrs. The media were removed and replaced with 150 μL MTT and then incubated for 15 min. Finally, the OD was measured at 570 nm in a microplate reader (BMGLABTECH^®^FLUOstar Omega, Ortenberg, Germany) [38].

#### 3.2.3. In Vivo Studies

##### Animals

Female Sprague-Dawley rats (age, 6 to 8 weeks/body weight 250 to 350 g) were used in both normal (used in the imaging studies) and pneumonic models. Individually housed rats were given free access to food and water in ventilated cages. The hazard ratio was used to estimate the group sizes. At the end of the experiment, CO_2_ euthanasia was used. Changes in body temperature and body weight, as well as pallor, nose bleeding, lack of reactivity, inactivity, instability, or abnormal breathing, were monitored every 12 h for 7 days to assess the disease progression.

##### Ethics

Animals were maintained and handled in accordance with the Guidelines of the Animal Care and Use Committee ((ACUC), Faculty of Science, Alexandria University, AU/04/22/04/13/06/02). The current study was performed in accordance with the in vivo experiments (ARRIVE) guidelines for reporting animal research [39].

##### Endotracheal Aerosolization of **3a**-ZnO, **3b**-ZnO and **3c**-ZnO in Rats

To achieve deep sedation, rats were anesthetized with 3–5% isoflurane. Animals were put in a supine posture on a rodent work stand (Hallowell EMC, Pittsfield, MA, USA) at a 45° angle. For rat endotracheal aerosolization, a MicroSprayer-syringe assembly was used (Penn-Century, Inc., Wyndmoor, PA, USA). Endotracheal aerosolization was used to deliver a predetermined amount of 1,2,3-triazole-sulphadiazine-ZnO hybrids suspensions (50 µL), and then the rats were kept in place for 10 s to allow for complete drug inhaling [40].

##### Radiolabeling of **3a**-ZnO, **3b**-ZnO and **3c**-ZnO for Imaging Studies

Electrophilic aromatic substitution on the tyrosine residues was used to radioiodinate 1,2,3-triazole-sulphadiazine-ZnO hybrids. For this, 1 mg/mL 1,2,3-triazole-sulphadiazine-ZnO hybrid solutions were incubated with [^124I^]NaI (Perkin Elmer, Inc., Waltham, MA, USA) for 2 h in 50 µL of 0.2 M sodium acetate buffer solution (pH 5.5) at 25 °C in the presence of Iodo-beads (Thermo Fisher Scientific, Waltham, MA, USA) according to Simone et al. [40].

##### Imaging Studies of 1,2,3-Triazole-sulphadiazine-ZnO Hybrids in Uninfected Rats

After the endotracheal aerosolization of the labeled 1,2,3-triazole-sulphadiazine-ZnO hybrids, normal (uninfected) animals were placed in a computerized tomography (CT) and positron emission tomography (PET) after administration (1 and 24 h post-administration). A CT scan (X-ray energy, 40 kV; intensity, 140 A) was carried out after each PET capture to allow for subsequent attenuation correction in image reconstruction and unambiguous radioactive signal localization. Images were evaluated using MOD analysis software (version 3.4; PMOD Technologies, Ltd., Zürich, Switzerland) after reconstruction. The concentration of radioactivity in the lungs was assessed for each component and time point by manually delineating volumes of interest (VOIs) in the whole lungs [40].

##### Pneumonia Model

A bacterial suspension was made from a second subculture that was cultured for less than 20 h to generate 107 CFU/mL in 3% (*w*/*v*) mucin. Serial dilution was used to confirm the inoculum density. Rats were anesthetized mildly with vaporized isoflurane (3–5%) then an intranasal injection with 50 µL bacterial suspensions was performed to induce pneumonia in rats [41].

(a) Bacterial Load Assessment

The lung lobes were removed aseptically and homogenized in sterile phosphate buffer saline (PBS) once the lungs were fully perfused with no visual blood. The homogenized tissues were then serially diluted and plated onto HIA plates to determine bacterial load (CFU per tissue weight) [41].

(b) Histopathological Studies

Light Microscopic Examination: Lungs were retrieved as soon as possible following euthanasia and deposited in 10% formalin. All lung tissues were fixed for at least 48 h. Sections of fixed tissues were cut and stained with hematoxylin and eosin (H&E) after being embedded in paraffin [41].

Transmission Electron Microscopic Examination: Lung tissues were preserved in 2.5% glutaraldehyde, acetone, and embedding solution blocks, and sliced into ultrathin slices of 50 to 60 nanometers. After staining with 3% uranium citrate lead acetic acid, the ultrastructure was examined using a transmission electron microscope (JEM-100 CX Joel) [42].

## 4. Conclusions

Sulphadiazine-ZnO hybrids were tested in vitro and in vivo against metallo-β-lactamases (MBLs) producing *Klebsiella pneumoniae*. Docking studies against NDM-1 and IMP-1 MBLs revealed that the synthesized compounds can inhibit both MBL enzymes in comparison to the reference drug, while the enzyme assay revealed that the synthesized sulphadiazine-ZnO hybrids inhibited the MBLs enzymes both competitively and noncompetitively. Compound **3b**-ZnO showed the highest antibacterial activity against MBLs producing *Klebsiella pneumoniae* with potent safety in a normal lung cell line (BEAS-2Bs cell line). Moreover, the [^124I^]**3b**-ZnO biodistribution in the lungs of uninfected rats revealed the detectable presence of the tested hybrid within the rats’ lungs after 24 h of endotracheal aerosolization with a remarkable residence duration reaching 85.3%. The histopathological investigations confirmed that **3b**-ZnO has significant activity in controlling bacterial pneumonia infection in rats. The above-mentioned results may pave the way in suggesting **3b**-ZnO as a potent pneumonia drug and MBLs enzyme inhibitor with significant antibacterial activity, residence duration, and potent safety.

## Figures and Tables

**Table 1:** Binding scores and binding interactions of the investigated compounds with IMP-1 active site.

Compound	IMP-1 Binding Score (Kcal/mol)	Hydrogen Bond Interactions	Hydrophobic Interactions
Captopril	−5.68	Lys179 and Asn185	His215
**3a**	−7.37	Lys179	Tyr181 and His215
**3b**	−7.45	Lys179 and Gly184	His215
**3c**	−7.15	Lys179	His215
ZnO	−12.71	-	-

**Table 2:** Binding scores and binding interactions of the investigated compounds with NDM-1 active site. Biological evaluations.

Compound	NDM-1 Binding Score (Kcal/mol)	Hydrogen Bond Interactions	Hydrophobic Interactions
Captopril	−5.64	Asn220	His250
**3a**	−5.93	Asn220	His250
**3b**	−5.57	Asn220	His250
**3c**	−5.53	-	His250
ZnO	−13.11	-	-

**Table 3:** Prevalence of NDM and IPM genes in the Kp tested strains.

Bacterial Strain	NDM	IPM
*K. pneumoniae* ATCC 13883	−	−
*K. pneumoniae* strain 1	+	+
*K. pneumoniae* strain 2	−	−
*K. pneumoniae* strain 3	−	−
*K. pneumoniae* strain 4	−	−
*K. pneumoniae* strain 5	+	+
*K. pneumoniae* strain 6	−	−
*K. pneumoniae* strain 7	−	−
*K. pneumoniae* strain 8	+	+
*K. pneumoniae* strain 9	−	−

**Table 4:** Enzyme inhibition essay.

Tested Compounds	% Inhibition		NDM-1		IPM-1	
	NDM-1 (5 µM)	IPM-1 (5 µM)	Kic (µM)	Kiuc (µM)	Kic (µM)	Kiuc (µM)
**3a**	71.2	75.0	9.6	6.0	11.8	7.7
**3b**	68.7	75.4	9.0	6.0	11.4	7.6
**3c**	64.3	72.9	8.5	3.9	10.9	5.1
**3a**-ZnO	90.0	95.0	14.3	6.4	17.2	8.7
**3b**-ZnO	87.0	95.5	14.7	6.5	17.8	9.4
**3c**-ZnO	85.0	90.0	10.6	4.7	15.3	8.6

Kic: inhibition constant for the competitive inhibition mode, Kiuc: inhibition constant for the uncompetitive inhibition mode.

**Table 5:** Antibacterial activity of the synthesized compounds vs. their hybrids.

Tested Compounds	Kp 1		Kp 5		Kp 8	
	IZ (mm)	MIC (µg/mL)	IZ (mm)	MIC (µg/mL)	IZ (mm)	MIC (µg/mL)
**3a**	9	128	7	256	8	128
**3b**	10	128	7	256	10	128
**3c**	6	256	6	256	6	256
ZnO	25	16	18	32	20	16
**3a**-ZnO	37	4	30	8	32	8
**3b**-ZnO	43	2	35	4	40	2
**3c**-ZnO	33	8	25	16	28	16

## Data Availability

The data presented in this study are available on request from the corresponding authors.

## Supplementary Materials

The following supporting information can be downloaded at: https://www.mdpi.com/article/10.3390/antibiotics11070916/s1, Figure S1. 1H NMR Spectrum of the 1,2,3-triazole-sulfoamide molecular conjugates **3a**. Figure S2. 13C NMR Spectrum of the 1,2,3-triazole-sulfoamide molecular conjugates **3a**. Figure S3. Photomicrograph of rat lung infected withKp5 and given endotracheal aerosolization various preparations of 1,2,3-triazole-sulphadiazine-ZnO hybrids as treatments, 96 h post infection (A) Negative control group. (B) Positive control (C) **3c**-ZnO (D) **3a**-ZnO and (E) **3b**-ZnO. In which black arrows refer congestion; red arrows refer to blood vessel dilated in both groups (D,E); TB refers to terminal bronchioles which was dilated in groups of (C,D) H&E stain (Magnification: 100X). Figure S4. Photomicrograph of rat lung infected withKp5 and given endotracheal aerosolization various preparations of 1,2,3-triazole-sulphadiazine-ZnO hybrids as treatments, 96 h post infection (A) Negative control group. (B) Positive control (C) **3c**-ZnO (D) **3a**-ZnO and (E) **3b**-ZnO. In which yellow arrows refer to signs of edema; H&E stain (Magnification: 100×).
