# Supplementary material for: Novel 1,2,3-Triazole-sulphadiazine-ZnO Hybrids as Potent Antimicrobial Agents against Carbapenem Resistant Bacteria
Source: Antibiotics (Basel). 2022 Jul 7;11(7):916. doi: 10.3390/antibiotics11070916 (PMC9312158; doi:10.3390/antibiotics11070916)
Supplement: Supplementary file 1 [file antibiotics-11-00916-s001.zip › antibiotics-1801590-supplementary.pdf]

Novel 1,2,3-triazole-sulphadiazine-ZnO hybrids as potent antimicrobial agents against carbapenem resistant bacteria

Faizah S. Aljohani[1,*], Nadjet Rezki[1], Mohamed R. Aouad[1], Mohamed Hagar[2], Basant A. Bakr[3], Marwa M. Shaaban[4], and Bassma H. Elwakil[5,*]

[1]Department of Chemistry, College of Science, Taibah University, Al-Madinah Al-Munawarah 30002, Saudi Arabia

[2]Department of Chemistry, Faculty of Science, Alexandria University, Alexandria 21321, Egypt

[3]Department of Zoology, Faculty of Science, Alexandria University, Alexandria 21321, Egypt

[4]Department of Pharmaceutical Chemistry, Faculty of Pharmacy, Alexandria University, Alexandria 21521, Egypt

[5]Department of Medical Laboratory Technology, Faculty of Applied Health Sciences Technology,Pharos University in Alexandria, Alexandria 21321 , Egypt

*Correspondence: m.sfm@hotmail.com (F.S.A.); bassma.hassna@pua.edu.eg (B.H.E)

Chemistry

Synthesis of 1,2,3-triazoles bearing sulfa-drug 3a-c

The click products **3a-c** were prepared in accordance with our previous work [25].

A solution of copper sulphate (0.10 g) and sodium ascorbate (0.15 g) in water (10 mL) was added with stirring to a solution of propargyl alcohol (**1**) (1 mmol) in DMSO (10 mL). The reaction mixture was then stirred at room temperature for 6-10 hours with the suitable sulfa azide **2a-c** (1 mmol). The reaction was monitored by TLC (hexane-ethyl acetate), and once complete, cold water was added to the mixture. Filtration was used to collect the precipitate, which was then washed with saturated ammonium chloride solution and recrystallized from ethanol/DMF to obtain the required 1,2,3-triazoles **3a-c**.

Characterization of 4-(4-(hydroxymethyl)-1H-1,2,3-triazol-1-yl)-N-(pyrimidin-2-yl)benzenesulfonamide (3a). It was obtained as white crystal in 88 % yield; Mp: 185-186 °C. IR

(KBr) ύ$_{max}$/cm^{-1}: 1582 (C=C), 1639 (C=N), 2899, 2947 (Al.C-H), 3b6 (Ar.C-H), 33c(NH), 3487 cm^{-1} (OH). ^{1}H NMR (DMSO-d_6, 400 MHz):δ_H = 12.10 (1H, s, N**H**SO_2), 8.79 (1H, s, C**H**-1,2,3-triazole), 8.53 (2H, bs, Ar-**H**), 8.15 (4H, bs, Ph-**H**), 7.07 (1H, bs, Ar-**H**), 5.41 (1H, s, O**H**), 4.61 (2H, s, C$\mathbf{H_2}$$NH_2$). ^{13}C NMR (DMSO-$d_6$, 100 MHz):$\delta_C$ = 158.95, 158.85, 149.93, 144.56, 138.75, 127.99, 127.78, 121.65, 121.58, 120.42, 120.27 (**C**=N, Ar-**C**), 55.16 (**C**H_2). Calculated for $C_{13}H_{12}N_6O_3S$: C: 46.98; H: 3.64; N: 25.29. Found: C: 46.69; H: 3.35; N: 25.08. HRMS (ESI): 332.0443 [M^+].

Characterization of 4-(4-(hydroxymethyl)-1H-1,2,3-triazol-1-yl)-N-(pyridin-2-yl)benzenesulfonamide (3b).

It was obtained as yellow crystal in 86 % yield; Mp: 223-224 °C. IR (KBr) ύ$_{max}$/cm^{-1}: 1577 (C=C), 1642 (C=N), 2889, 2956 (Al.C-H), 3082 (Ar.C-H), 3323(NH), 3498 cm^{-1} (OH). ^{1}H NMR (DMSO-d_6, 400 MHz):δ_H = 12.58 (1H, s, N**H**SO_2), 8.80 (1H, s, C**H**-1,2,3-triazole), 8.13 (4H, bs, Ph-**H**), 6.87-7.20 (4H, m, Ar-**H**), 5.38 (1H, s, O**H**), 4.62 (2H, s, C$\mathbf{H_2}$$NH_2$). ^{13}C NMR (DMSO-$d_6$, 100 MHz):$\delta_C$ = 169.90, 163.73, 158.90, 157.86, 149.82, 147.99, 139.26, 132.93, 129.40, 128.47, 121.21, 120.12, 119.99 (**C**=N, Ar-**C**), 55.31 (**C**H_2). Calculated for $C_{14}H_{13}N_5O_3S$: C: 50.75; H: 3.95; N: 21.14. Found: C: 50.38; H: 3.55; N: 21.47. HRMS (ESI): 331.0598 [M^+].

Characterization of N-(diaminomethylene)-4-(4-(hydroxymethyl)-1H-1,2,3-triazol-1-yl)benzenesulfonamide (3c). It was obtained as yellow pale crystal in 90 % yield; Mp: 254-256 °C. IR (KBr) ύ$_{max}$/cm^{-1}: 1577 (C=C), 1642 (C=N), 2889, 2956 (Al.C-H), 3082 (Ar.C-H), 3319-3376 (NH_2), 3509 cm^{-1} (OH). ^{1}H NMR (DMSO-d_6, 400 MHz):δ_H = 8.78 (1H, s, C**H**-1,2,3-triazole), 8.05 (2H, d, J = 4Hz, Ar-**H**), 7.93 (2H, d, J = 4Hz, Ar-H), 6.90 (4H, s, 2xN$\mathbf{H_2}$), 5.45 (1H, s, O**H**), 4.62 (2H, s, C$\mathbf{H_2}$). ^{13}C NMR (DMSO-d_6, 100 MHz):δ_C = 158.31, 149.42, 144.22, 144.02, 138.58, 127.75, 121.87, 120.59, 119.99 (**C**=N, Ar-**C**), 55.54 (**C**H_2). Calculated for $C_{10}H_{12}N_6O_3S$: C: 40.54; H: 4.08; N: 28.36. Found: C: 40.89; H: 4.37; N: 28.59. HRMS (ESI): 296.0338 [M^+].

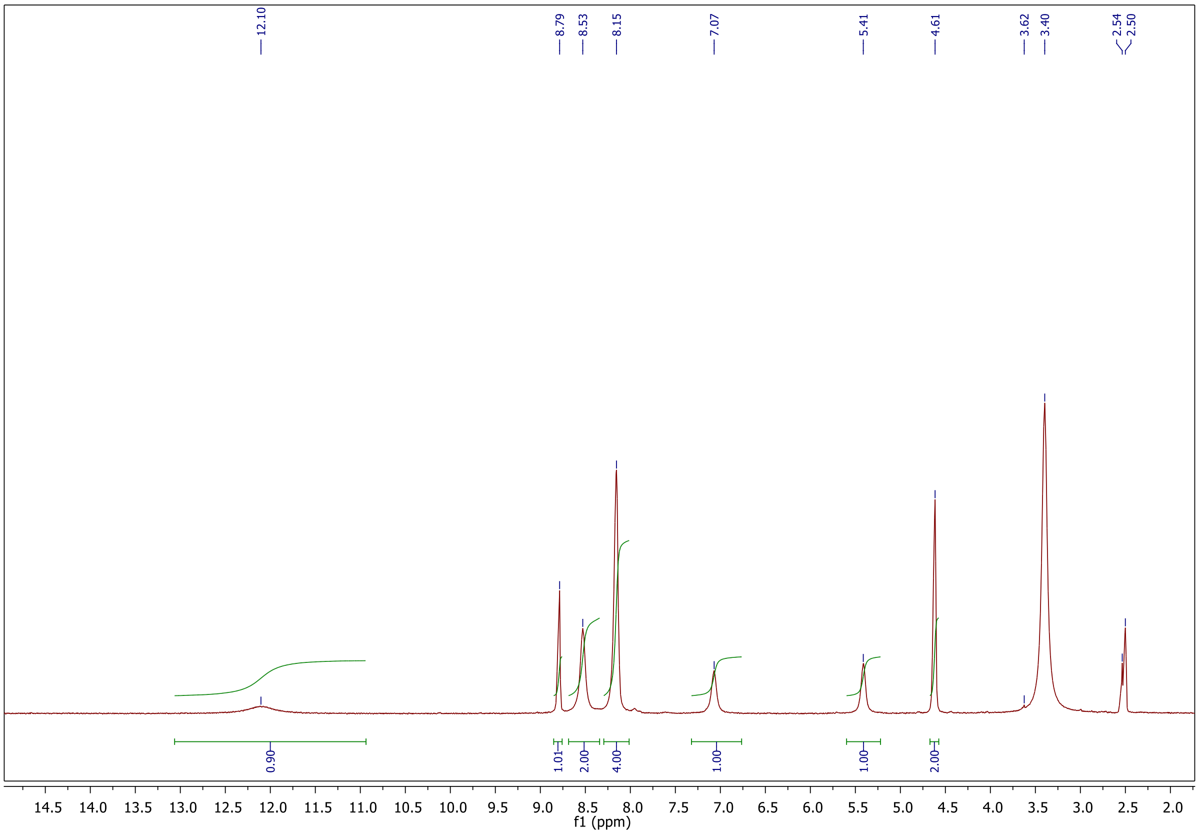

Figure S1. ^{1}H NMR Spectrum of the 1,2,3-triazole-sulfoamide molecular conjugates **3a.**

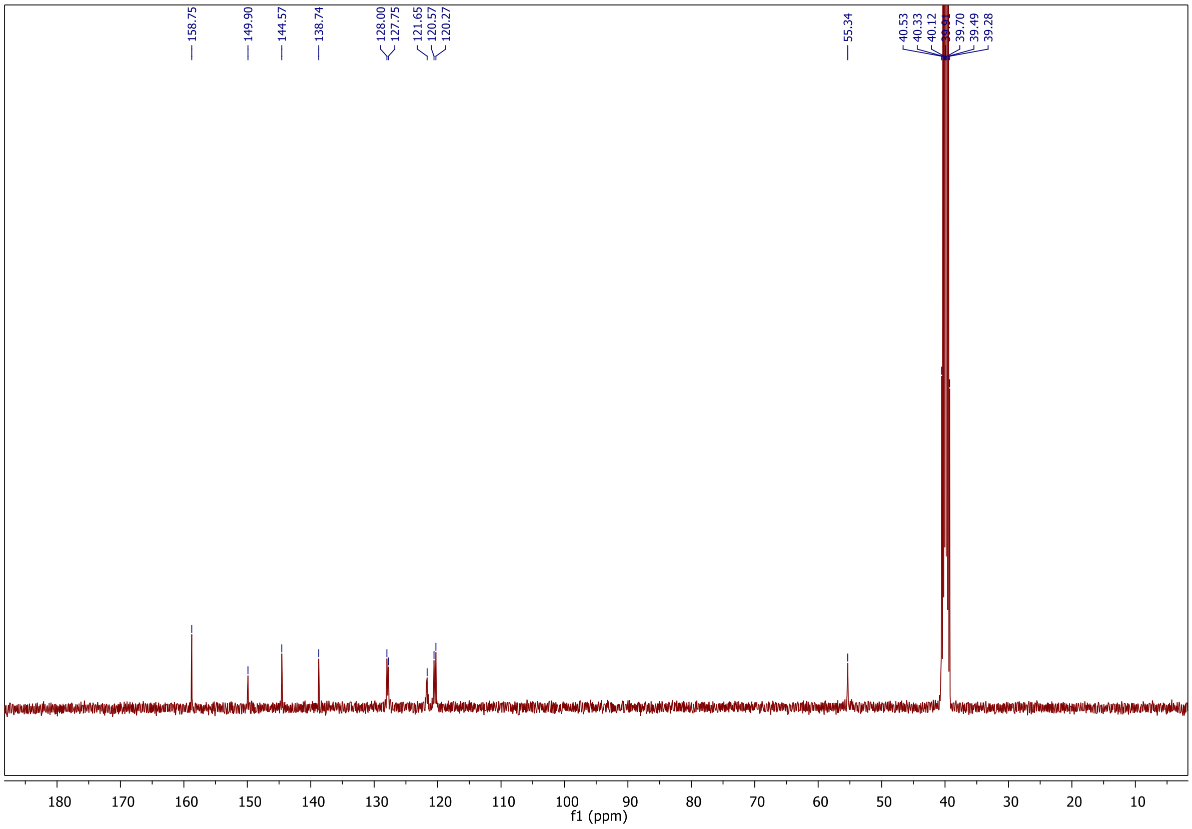

Figure S2. ^{13}C NMR Spectrum of the 1,2,3-triazole-sulfoamide molecular conjugates **3a.**

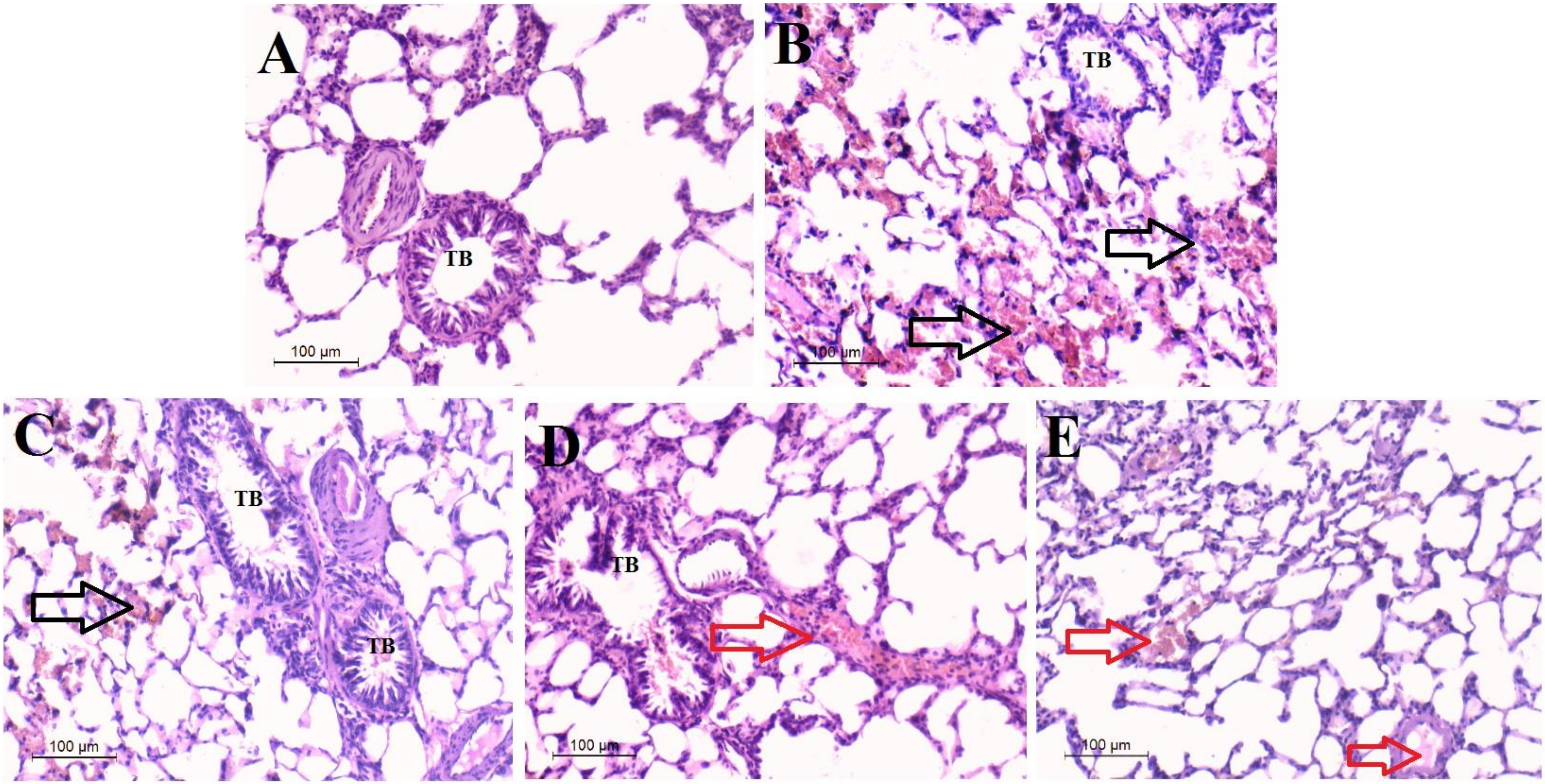

Figure S3. Photomicrograph of rat lung infected withKp5 and given endotracheal aerosolization various preparations of 1,2,3-triazole-sulphadiazine-ZnO hybrids as treatments, 96 h post infection (A) Negative control group. (B) Positive control (C) 3c-ZnO (D) 3a-ZnO and (E) 3b-ZnO. In which black arrows refer congestion ; red arrows refer to blood vessel dilated in both groups(D,E) ;TB refers to terminal bronchioles which was dilated in groups of (C,D) H&E stain (Magnification: 100X)

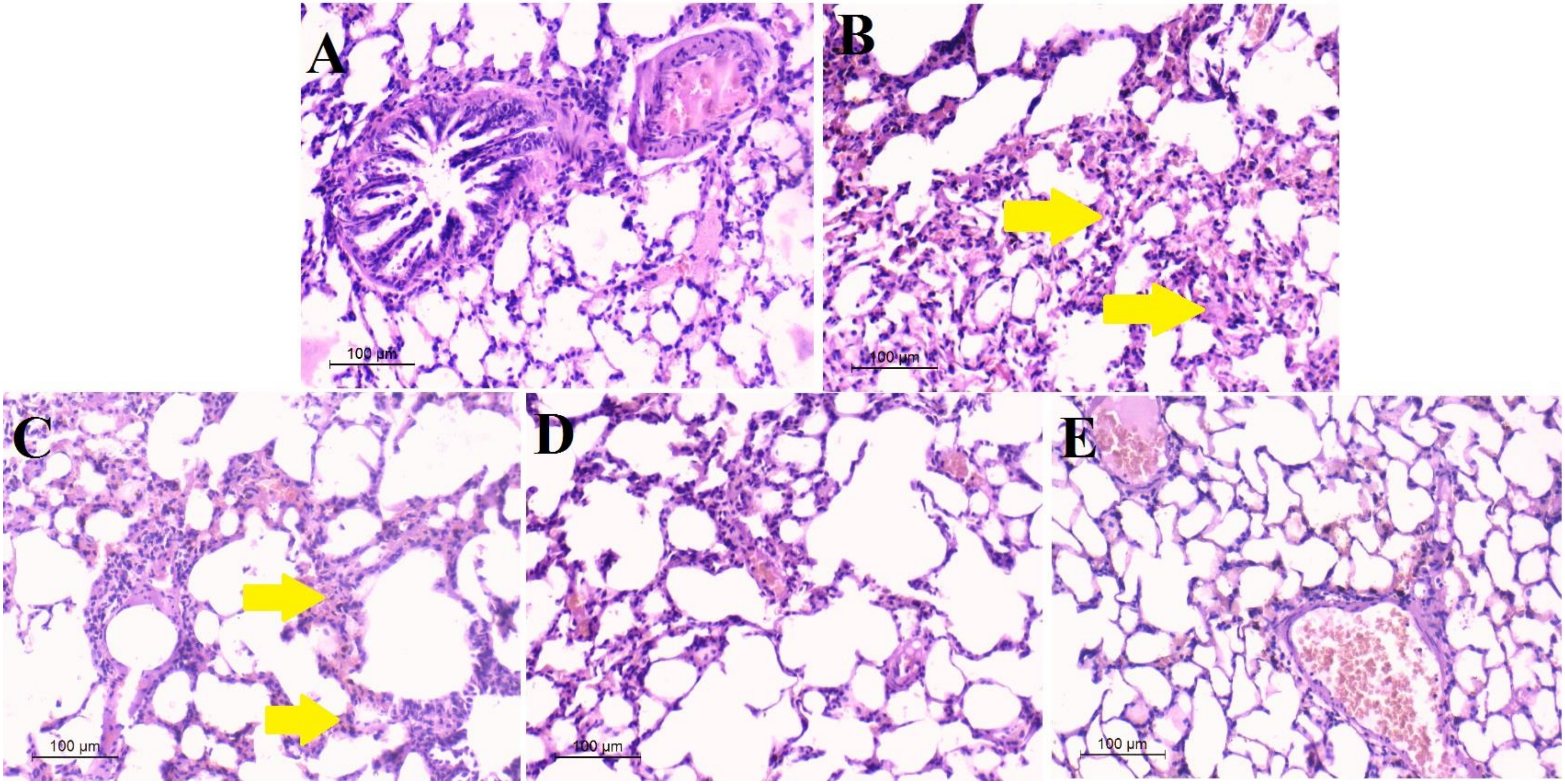

Figure S4. Photomicrograph of rat lung infected withKp5 and given endotracheal aerosolization various preparations of 1,2,3-triazole-sulphadiazine-ZnO hybrids as treatments, 96 h post infection (A) Negative control group. (B) Positive control (C) 3c-ZnO (D) 3a-ZnO and (E) 3b-ZnO. In which yellow arrows refer to signs of edema; H&E stain (Magnification: 100X)
